# Disabled-2: a protein up-regulated by high molecular weight hyaluronan has both tumor promoting and tumor suppressor roles in ovarian cancer

**DOI:** 10.1007/s00018-023-04972-9

**Published:** 2023-10-10

**Authors:** Zoe K. Price, Noor A. Lokman, Mai Sugiyama, Yoshihiro Koya, Masato Yoshihara, Martin K. Oehler, Hiroaki Kajiyama, Carmela Ricciardelli

**Affiliations:** 1 Reproductive Cancer Group, Discipline of Obstetrics and Gynaecology, Adelaide Medical School, Robinson Research Institute, Adelaide Health and Medical Sciences Building, The University of Adelaide, Level 5, North Terrace, Adelaide, SA 5000 Australia; 2 Department of Obstetrics and Gynecology, Nagoya University Graduate School of Medicine, Nagoya, Japan; 3 Department of Obstetrics and Gynecology Collaborative Research, Bell Research Center, Nagoya University Graduate School of Medicine, Nagoya, Japan; 4 Department of Gynaecological Oncology, Royal Adelaide Hospital, Adelaide, 5000 Australia

**Keywords:** Disabled-2, Hyaluronan, Notch3, Metastasis, Prognosis, EMT

## Abstract

Although the pro-tumorigenic functions of hyaluronan (HA) are well documented there is limited information on the effects and targets of different molecular weight HA. Here, we investigated the effects of 27 kDa, 183 kDa and 1000 kDa HA on ES-2 ovarian cancer cells overexpressing the stem cell associated protein, Notch3. 1000 kDA HA promoted spheroid formation in ES-2 cells mixed with ES-2 overexpressing Notch3 (1:3). We report disabled-2 (DAB2) as a novel protein regulated by 1000 kDa HA and further investigated its role in ovarian cancer. *DAB2* was downregulated in ovarian cancer compared to normal tissues but increased in metastatic ovarian tumors compared to primary tumors. High *DAB2* expression was associated with poor patient outcome and positively correlated with HA synthesis enzyme *HAS2*, HA receptor *CD44* and EMT and macrophage markers. Stromal DAB2 immunostaining was significantly increased in matched ovarian cancer tissues at relapse compared to diagnosis and associated with reduced survival. The proportion of DAB2 positive macrophages was significantly increased in metastatic ovarian cancer tissues compared to primary cancers. However, DAB2 overexpression significantly reduced invasion by both A2780 and OVCAR3 cells in vivo. Our research identifies a novel relationship between HA signalling, Notch3 and DAB2. We highlight a complex relationship of both pro-tumorigenic and tumor suppressive functions of DAB2 in ovarian cancer. Our findings highlight that DAB2 has a direct tumor suppressive role on ovarian cancer cells. The pro-tumorigenic role of DAB2 may be mediated by tumour associated macrophages and requires further investigation.

**Supplementary Information:**

The online version contains supplementary material available at 10.1007/s00018-023-04972-9.

## Introduction

In 2020, ovarian cancer was the fourth most lethal cancer for women in countries with a very high health development index [62]. Primary treatment for ovarian cancer consists of a combination of debulking surgery and chemotherapy. High grade serous ovarian cancer (HGSOC) is the most prominent subtype, accounting for approximately 70% of cases. It is initially chemotherapy-responsive, but unfortunately 75% of patients experience a recurrence and eventually develop chemotherapy resistance [10].

Hyaluronan (HA), a glycosaminoglycan, is a signaling molecule in cell proliferation, embryogenesis, wound healing and inflammation [19, 57, 65, 81]. It is a key component of the tumor extracellular matrix (ECM) and is commonly upregulated in cancer. HA primarily interacts with receptor CD44 to initiate a range of pro-cancer signals including enhanced therapy resistance, cell proliferation, cell migration, cell invasion and activation of stem cell signaling [38, 44, 79]. Biologically, HA is present as different molecular weight polymers, low-molecular weight HA (LMW-HA) is pro-inflammatory and pro-angiogenic whilst high molecular weight HA (HMW-HA) is anti-inflammatory and anti-angiogenic [18, 40, 57]. Increasing molecular weight HA enhances binding affinity for receptor CD44 [27, 71, 76]. Furthermore, increasing molecular weight HA enhance clustering of CD44 at the plasma membrane [27, 71, 76]. Structurally, HA polymers below 150 kDa have a rod like formation, polymers over 250 kDa a coil like formation and polymers between 150 and 250 kDa have an intermediate structure with both coil and rod features [70].

The effect of HA in cancer is widely studied, however the role of different molecular weight HA in cancer is poorly understood. In ovarian cancer, HMW-HA enhances cell migration and therapy resistance [4, 5]. HA is upregulated in HGSOC patients with chemotherapy resistant disease and promotes therapy resistance through expression of ABC transporters *ABCB3, ABCC1, ABCC2,* and *ABCC3* [37, 58]. HA correlates with poor prognosis in cancers of ovary, breast, prostate, stomach and bowel [32, 63].

The Notch3 signaling pathway is involved in cell fate, cell differentiation and cell proliferation [74]. Activation of Notch3 signaling upon interaction with co-activator ligands causes cleavage of Notch3 intracellular domain (NICD3), a transcription factor [74]. In ovarian cancer, Notch3 correlates with reduced OS, PFS, metastasis and therapy resistance [33, 53]. Furthermore, Notch3 is known for maintaining cancer stem cell (CSC) markers and function in ovarian cancer including colony and spheroid formation, therapy resistance and in vivo tumorigenicity [33, 53].

The main mechanism of ovarian cancer metastasis involves the formation of spheroids in ascites in advanced stage patients, allowing the transport of ovarian cancer cells to the peritoneum, the primary site of ovarian cancer metastasis [67]. CSC have been detected in ovarian cancer and are shown to have increased spheroid formation and are involved in ovarian cancer progression [67]. In this study, we overexpressed NICD3 in an ovarian cancer cell line with low NOTCH3 expression (ES-2) to simulate a stem-like model. We assessed the effects of three different molecular weight HA (27 kDa, 183 kDa and 1000 kDa) on spheroid formation. Proteins differentially regulated by the different HA were identified by mass spectrometry. We found disabled-2 (DAB2) protein levels were up-regulated by 1000 kDa HA in ES-2 ovarian cancer cells mixed with ES-2 cells overexpressing NICD3 (ratio 1:3) and two HGSOC cell lines (OVCAR3 and OV90). DAB2 is an endocytic adaptor protein involved in clathrin mediated endocytosis and is frequently downregulated in cancer [15, 55]. Loss of DAB2 in cancer activates Wnt and MAPK signaling, promoting EMT, cell migration and tumor formation [55]. We utilized online databases and independent tissue cohorts to further examine the relationship between DAB2 and ovarian cancer progression. We also evaluated the functional roles of DAB2 overexpression in vitro (cell proliferation, migration, invasion, therapy resistance and spheroid formation) and in vivo (cell invasion).

## Materials and methods

### Cell culture

The human ovarian cancer cell line A2780 was purchased from European Collection of Authenticated Cell cultures (ECACC, Salisbury, UK). OVCAR3 and OV90 cells were purchased from American Type Culture Collection (ATCC, Manassas, VA, USA). ES-2 cells were generously provided by Dr H. Albrecht (University of South Australia). All cell lines were verified by short tandem repeat (STR) testing in 2021 (Promega GenePrint^®^10; Griffith University DNA sequencing facility, QLD, Australia). A2780, ES-2 and OVCAR3 cell lines were maintained in RPMI-1640 media (11,875,093, Life Technologies, Carlsbad, CA, USA) supplemented with 10% fetal bovine serum (FBS) (Bovogen Biologicals, Melbourne, Vic, Australia) and antibiotics (100U penicillin G, 100 µg/mL streptomycin sulphate and 0.25 µg/mL amphotericin B, Sigma-Aldrich, St Louis, MO, USA) and maintained at 37 °C in 5% CO_2_ environment.

### Viral transduction

pQCVIP vector (TAKARA, Osaka, Japan) empty (Rv-Ctrl) or encoding NICD3 (Rv-NICD3) (Q9UM47, aa1666-2321) was transfected into HEK-293T cells in combination with the pVPack-GP and pVPack-Amphop vectors (Stratagene, Tokyo, Japan) using Lipofectamine 2000 (Invitrogen, Carlsbad, CA, USA). 48 h after transfection, the supernatants were added to ES-2 cells with polybrene (2 µg/mL, Sigma-Aldrich), and ES-2 cells with empty vector (ES-2-Rv-Ctrl) or NICD3 cDNA (ES-2-Rv-NICD3) were selected by puromycin selection (1 µg/mL, Sigma-Aldrich) for 3 days.

Lentiviral vectors containing pGenLenti-DAB2-IRES-puro (Lv-DAB2, clone ID: OHu26906, GenScript, Piscataway, New Jersey, USA) or control pLVX-EGFP-IRES-puro (Lv-Ctrl, #128652, a gift from Robert Sobol, Addgene, Watertown, MA, USA [13]) were generated by Gene Silencing and Expression Core Facility (The University of Adelaide, Adelaide, SA). A2780 and OVCAR3 cells (1 × 10^5^ cells/well) in 24 well plates were cultured 5 h with polybrene (8 µg/mL, Sigma-Aldrich) before transduction with lentivirus (Lv-Ctrl or Lv-DAB2) at multiplicity of infection (MOI) 1.5 and 3 respectively. After 7 days, OVCAR3 and A2780 cells were selected with 0.5 and 1.2 µg/mL puromycin (Sigma-Aldrich) respectively for 1 week.

### Spheroid assay

ES-2, ES-2-Rv-Ctrl, ES-2-Rv-NICD3 cells and a population of ES-2 cells combined with ES-2-Rv-NICD3 (ES-2:ES-2-Rv-NICD3, 1:3) were plated at 10,000 cells/well on 24 well plates coated with polyHema (30 mg/mL, Sigma-Aldrich) with or without 1000 kDa HA (50 µg/mL, Contipro Inc., Dolní Dobrouč, Czechia). After 72 h, spheroids were imaged by light microscopy (Olympus IX-71, 5 images/well) and spheroid area was quantitated using FIJI (FIJI 2, version 2, Cambridge Astronomical Survey Unit). OV90 and OVCAR3 cells were plated at 50,000 cells/well with or without 1000 kDa HA (50 µg/mL) for 72 h before imaging by EVOS light microscope (Thermo Fisher Scientific, Waltham, MA, USA), 5 images/well. A2780 and OVCAR3 (Lv-Ctrl or Lv-DAB2, 50,000 cells/well) were cultured 3 and 5 days respectively before imaging.

### Cell metabolism and carboplatin response

ES-2-Rv-Ctrl and ES-2-Rv-NICD3 cells (1000 cells/well, quadruplicate, 96 well plates) were cultured for 96 h and assessed by MTS assay (Promega, Madison, MA, USA) as per manufacturer’s instructions. Cell metabolism of OVCAR3 and A2780 cells (Lv-Ctrl or Lv-DAB2, 7,500 cells/well, 96 well plates) was assessed at 24 h, 48 h, 72 h and 96 h by MTT (Sigma-Aldrich) in quadruplicate. Carboplatin response assays were performed for OVCAR3 and A2780 (Lv-Ctrl or Lv-DAB2) cells plated at 7,500 and 5,000 cells/well respectively as previously described [31].

### Quantitative RT-PCR

RNA was isolated from ES-2-Rv-Ctrl and ES-2-Rv-NICD3 (3 wells/replicate) spheroids with Qiagen RNeasy mini isolation kit (Qiagen, Hilden, Germany) as per manufacturer’s instructions. 200 ng of RNA was reverse transcribed using the high-capacity cDNA reverse transcriptase kit (Thermo Fisher Scientific) as per manufacturer’s instructions. qRT-PCR reactions were performed on triplicate cDNA samples with TaqMan^®^ primers (ID: 4453320, Thermo Fisher Scientific) (Supplementary Table. 1) as described previously [37]. Expression was normalized *GAPDH* and calibrator with the 2^−∆∆CT^ method.

### Liquid chromatography with tandem mass spectrometry (LC–MS/MS)

Based on spheroid assay results ES-2 cells were combined with ES-2-Rv-NICD3 cells at a ratio of 1:3 and plated at 10,000 cells/well on 24 well polyHEMA plates with vehicle control, 27 kDa, 183 kDa or 1000 kDa HA (50 µg/mL, Contipro Inc.). Spheroid images were taken and quantitated as in the spheroid assay above. Spheroids were cultured 72 h and isolated with a 40 µM cell strainer (Pluriselect, Leipzig, Germany). Protein was extracted from duplicate samples for each treatment group as previously described and analysed using the Orbitrap Fusion (Thermo Fisher Scientific) [47]. All proteins detected in each sample were analysed and every value determined to fall beneath the level of detection sensitivity was substituted by the adjusted minimum detection number [52]. A fivefold change was set as the value for the geographic average between the paired groups as determined by the Mascot program (Version 2.6.0, Matrix Science Inc., Boston, MA, USA). This threshold for the protein abundance ratio confidently identified proteins with significantly altered expression in a previous study [29]. Differentially expressed proteins were identified using area and peptide spectrum match (PSM) counts for proteins with RStudio (Version 1.4.1103) and DEqMS package [82].

### Ovarian cancer online database analysis

Survival analysis for mean *DAB2* expression (probes: 201278_at, 201279_s_at, 201280_s_at, 210757_x_at, 240873_x_at; auto-select best cut-off) and PFS, post progression survival (PPS) and OS in HGSOC patients (grades 2 + 3, n = 321–483) was performed by Kaplan–Meier Plotter (kmplot.com) [30].

Co-expression analysis for *DAB2* was assessed in cBioPortal (v5.2.8; cbioportal.org) in datasets Cancer Cell Line Encyclopedia (CCLE) (Broad Institute, 2019, ovarian adenocarcinoma; n = 64) and Ovarian Serous Cystadenocarcinoma TCGA dataset (Firehose Legacy, n = 617) including RNA sequencing (= 307), Mass Spectrometry (n = 174), and microarray (n = 558).

GENT2 (http://gent2.appex.kr) analysis of GPL570 platform (HG-U133) microarray data for ovarian cancer patients (n = 1626 patients, n = 35 genomic spatial events (GSE) datasets) was assessed [54]. Individual samples were reviewed and specified as normal ovarian surface epithelium (OSE), fallopian tube (FT) and different subtypes of ovarian cancer (endometrial, mucinous, clear, HGSOC and low grade serous ovarian cancer (LGSOC)). Spearman correlation analysis was performed for datasets GSE40595 and GSE2109. GSE40595 dataset contained expression data for laser microdissected HGSOC tumor stroma and epithelium. GSE2109 dataset was accessed using the GEO2R platform (ncbi.nlm.nih.gov/geo/geo2r) for primary (n = 138), metastatic (n = 53), HGSOC primary (n = 68) and HGSOC metastatic (n = 36) ovarian tumors for mean *DAB2* expression (probes: 201278_at, 201279_s_at, 201280_s_at, 210757_x_at, 240873_x_at).

TIMER database (timer.cistrome.org) cibersort absolute analysis of M1 and M2 macrophage estimations was assessed for *DAB2* expression in TCGA ovarian cancer samples (n = 303).

### Patient tissue cohort

HGSOC tissue samples were collected with approval by the Royal Adelaide Hospital Human Ethics Committee (RAH protocol number 140101 and 060903). Tissue microarray (TMA) were assembled from paraffin embedded HGSOC patient samples diagnosed between 1988 and 2010 (n = 136) including primary (n = 87) and metastatic (n = 49) tissues (Supplementary Table. 2) [36]. Each patient tumor had duplicate or triplicate 1 mm diameter cores. Matched paraffin embedded HGSOC tissues at relapse and diagnosis (n = 4) were also assessed.

### Immunohistochemistry and immunofluorescence

Immunohistochemistry was performed as previously described with DAB2 rabbit monoclonal antibody (1/800, ab256524, Abcam, Cambridge, UK) [37]. Slides were scanned by NanoZoomer Digital Pathology System (Hamamatsu Photonics, Hamamatsu City, Japan). DAB2 immunostaining H-score in 3 tumor and stroma areas per core was quantitated using QuPath software (version 0.2.3) and maximum H-score was assessed in relation to patient prognosis and relapse [2]. For immunofluorescence, tissue sections from matching primary and metastatic cancers (n = 5) were incubated overnight at 4 °C with DAB2 (1/100, ab256524, Abcam) or CD68 mouse monoclonal antibody (1/400, ab955, Abcam). Positive cells were detected with α-rabbit-IgG (H + L) AlexaFluor™ Plus 594 (1/400, A23740, Invitrogen) or α-mouse-IgG (H + L) AlexaFluor™ Plus 488 (1/400, A23723, Invitrogen). Nuclei were visualized with DAPI (1.5 µg/mL, Molecular Probes, Life Technologies) as previously described [31]. Tissues were imaged with the BX50 epifluorescence microscope (40X objective, Olympus, Tokyo, Japan). Number of DAB2 positive ( +), CD68 + and double positive (DAB2 + CD68 +) cells in both tumor epithelium and tumor associated stroma areas were counted by two individual assessors in 3–8 sections/tissue. Number of positive cells was normalized to area of tumor epithelium or stroma (µm^2^).

### Western immunoblotting

ES-2 and ES-2:ES-2-Rv-NICD3 cells were plated at 2 × 10^5^ cells/well in 6 well plates and cultured 48 h before treatment with the hyaluronan synthesis inhibitor 4-methylumbelliferone (4-MU, 1 mM, Sigma-Aldrich) or vehicle control (PBS) for 24 h [37]. Protein lysates from monolayer and spheroids were prepared in RIPA buffer as described previously [59]. ES-2, A2780 and OVCAR3 cells were cultured to 80% confluency before protein isolation. 20 µg of protein was electrophoresed on 4–20% TGX gels (Bio-Rad, Hercules, CA, USA) and transferred overnight to PVDF membrane (GE healthcare, little Chalfont, England). Proteins were detected with DAB2 rabbit monoclonal antibody (1/2000, ab256524, Abcam), anti-rabbit IgG peroxidase conjugated antibody (1/4000, Sigma-Aldrich) and Amersham™ ECL™ Prime (Cytvia, Marlborough, MA, USA). Chemiluminescence was detected using the ChemiDoc™ Imaging System (Bio-Rad) and band intensity was calculated with ImageLab software (Bio-rad, version 6.1). *β-*actin monoclonal mouse antibody (1/5000, ab8226, Abcam) or GAPDH monoclonal mouse antibody (1/50,000, 60004-1-Ig, Proteintech®, Rosemont, IL, USA) was used as a loading control.

### Motility and invasion assay

Motility and invasion assays were performed as previously described for A2780 and OVCAR3 (Lv-Ctrl or Lv-DAB2) cells at 40,000 cells/well [35]. Cell motility and invasion was measured after 6 h.

### Chick chorioallantoic membrane (CAM) in vivo invasion assay

The CAM assay was performed as described previously [34]. A2780 (2 × 10^4^) or OVCAR3 (4 × 10^4^) cells (Lv-Ctrl or Lv-DAB2) were mixed with Matrigel (E6909, Sigma-Aldrich) and implanted on the CAM of day 11 chick embryos [34]. CAM tissue section (5 µm) were immunostained with Ki67 (1/600, clone SP6, cat # MA5-14520, Thermo Fisher Scientific) or ANXA2 (1/500, Clone 5, cat #. 610069, BD Bioscience, Franklin Lakes, NJ, USA) to visualise A2780 and OVCAR3 cells respectively. Slides were scanned by NanoZoomer (Hamamatsu Photonics) and OVCAR3 and A2780 cells invaded into the mesoderm area was measured using NDP view (NDP Scan software v2.2, Hamamatsu Photonics). Data was expressed as Ki67 or ANXA2 positive area (µm^2^/mm^2^ of mesoderm). The in vivo CAM assay was approved by the University of Adelaide ethics committee (Protocol number M-2018-087).

### Statistical analysis

For cell line experiments and online database analysis, un-paired Student’s T test and one-way ANOVA analysis were performed for normally distributed data (Prism 9 for MacOS, Version 9.3.1350, GraphPad, San Diego, CA, USA). The Mann–Whitney, Wilcoxon rank paired test or Kruskal–Wallis test were performed for data with non-normal distribution. Spearman correlation analysis of CCLE data was performed in RStudio (Version 1.4.1103, RStudio, Boston, MA, USA) using the Corrplot package. Kaplan–Meier survival analyses were performed with IBM^®^ SPSS^®^ Statistics software (Version 28.0.1.0, IBM^®^ Corporation, Armonk, NY, USA). A paired Student T test was applied to compare DAB2 immunostaining in relapse tissue and matched tissues at diagnosis. Statistical significance was accepted at p < 0.05. *p < 0.05; **p < 0.01; ***p < 0.001; ****p < 0.0001.

## Results

### 1000 kDa HA promotes spheroid formation of ES-2 ovarian cancer cells combined with ES-2 cells that overexpress NICD3

**Fig. 1:**
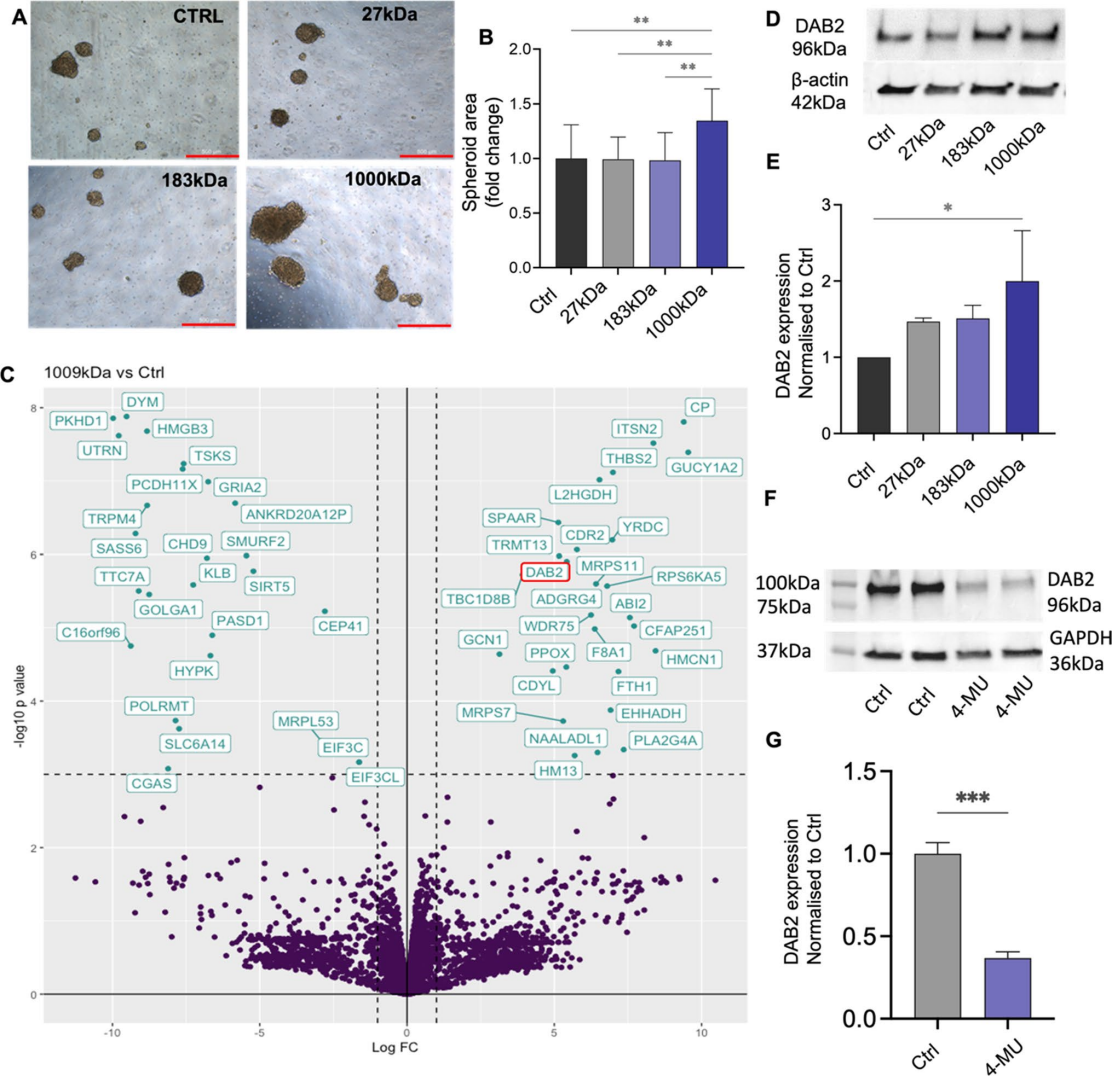
Effects of different molecular weight HA on ES-2:ES-2-Rv-NICD3 (1:3) spheroids. **A** Representative images of spheroids formed by combination ES-2:ES-2-Rv-NICD3 (1:3) cells treated with control (Ctrl), 27 kDa, 183 kDa or 1000 kDa HA (50 µg/mL) for 72 h. Scale bar 500 µm. **B** Quantitation of spheroid area compared to Ctrl (n = 3, experiments, n = 16, **p < 0.01, one-way ANOVA). **C** Volcano plot of differentially expressed proteins identified by LC–MS/MS in 1000 kDa HA treated ES-2:ES-2-Rv-NICD3 (1:3) spheroids compared to Ctrl. Highlighted proteins meet the cut offs of log_2_fold change 1 and p-value < 0.001. **D** Western blot analysis of DAB2 protein expression in ES-2:ES-2-NICD3 (1:3) spheroids treated with Ctrl, 27 kDa, 183 kDa or 1000 kDa HA for 72 h. **E** Quantitation of DAB2 protein expression normalized to control (n = 3, *p < 0.05, one-way ANOVA). **F** Western blot analysis of DAB2 protein expression in ES-2:ES-2-NICD3 (1:3) cells treated with PBS (Ctrl) or 4-MU (1 mM) for 24 h. **G** Quantitation of DAB2 protein expression normalized to control (n = 4, ***p = 0.0002, unpaired t test). Data presented as mean ± SD

We confirmed overexpression of NICD3 in ES-2 cells promoted stem cell-associated features including reduced cell metabolism indicative of reduced cell survival, significantly enhanced spheroid formation and enhanced expression of the stem cell related transcription factor *TWIST1* (1.28 fold, **p = 0.0096) (Supplementary Fig. S1A-C). ES-2 cells were selected due to their low expression of NOTCH3 (Supplementary Fig. S2A-B). Increased *NOTCH3* expression concominant with increased *NOTCH1* was observed in ES-2-Rv-NICD3 cells compared to ES-2-Rv-Ctrl (Supplementary Fig. S1D-E). Due to the cell–cell interactions normally required in NOTCH3 signalling and extracellular location of HA in normal biological conditions we hypothesised that combining WT and ES-2-Rv-NICD3 cells may enhance the stem associated features with NOTCH3 [51, 74]. Combination of ES-2-Rv-NICD3 cells with WT ES-2 cells at a ratio of 3:1 respectively significantly enhanced spheroid formation compared to control cells (Fig. S1F, p = 0.0021). We assessed the effects of HMW-HA (1000 kDa) on spheroid formation in the three ES-2 cell populations. 1000 kDa HA had no significant effect on spheroid formation in both WT ES-2 cells (Fig. S1G) and ES-2-Rv-NICD3 cells (Fig. S1H) compared to control but increased spheroid formation in ES-2 combined with ES-2-Rv-NICD3 cells (1:3) (Fig. S1I). 1000 kDa HA but not 27 kDa HA nor 183 kDa HA significantly enhanced spheroid formation by ES-2:ES-2-Rv-NICD3 (1:3) cells (Fig. 1A, B).

### DAB2 protein is up-regulated by 1000 kDa HA in ES-2 spheroids

LC–MS/MS was used to identify differentially expressed proteins in the ES-2:ES-2-Rv-NICD3 (1:3) spheroids treated with control, 27 kDa, 183 kDa or 1000 kDa HA (Fig. 1C and Supplementary Fig. S3A-B). Kegg pathway analysis of differentially expressed proteins (p < 0.05) in 1000 kDa HA treated ES-2:ES-2-Rv-NICD3 spheroids compared to control identified 9 proteins within the endocytosis pathway (Supplementary Fig. S3C-D). DAB2, an endocytic adaptor protein, was upregulated 5.2 fold (p = 1.25e−6) in the 1000 kDa HA treated spheroids (Fig. 1B) [16]. We validated DAB2 expression was enhanced in 1000 kDa HA treated ES-2:ES-2-Rv-NICD3 combination spheroids by western blot (Fig. 1D, E, twofold, p = 0.019). No significant effect on DAB2 expression was observed in response to 27 kDa or 183 kDa HA (Fig. 1D, E). To determine the involvement of NICD3 in the upregulation of DAB2 we compared DAB2 expression in ES-2:ES-2-Rv-NICD3 and ES-2 WT spheroids and also assessed the effects of 1000 kDa HA on DAB2 expression in ES-2 WT spheroids. No significant change in DAB2 expression was observed in 1000 kDa treated ES-2 WT spheroids compared to control (Supplementary Fig. S4A). However, DAB2 expression was increased in ES-2:ES-2-Rv-NICD3 compared to ES-2 WT spheroids (Supplementary Fig. S4D, fold change 1.53). Furthermore, we treated ES-2 and ES-2:ES-2-Rv-NICD3 combination cells with HA synthesis inhibitor 4-MU in both monolayer and spheroid culture. No effect on DAB2 expression was observed in ES-2 WT cells in both monolayer (Supplementary Fig. S4B) and spheroid culture (Supplementary Fig. S3C). DAB2 expression was decreased in 4MU treated ES-2:ES-2-Rv-NICD3 cells compared to control in both spheroid (Supplementary Fig. S4C, fold change 0.72) and monolayer (Fig. 1F. fold change = 0.36, p = 0.0002).

### DAB2 protein is up-regulated by 1000 kDa HA in HGSOC spheroids

**Fig. 2:**
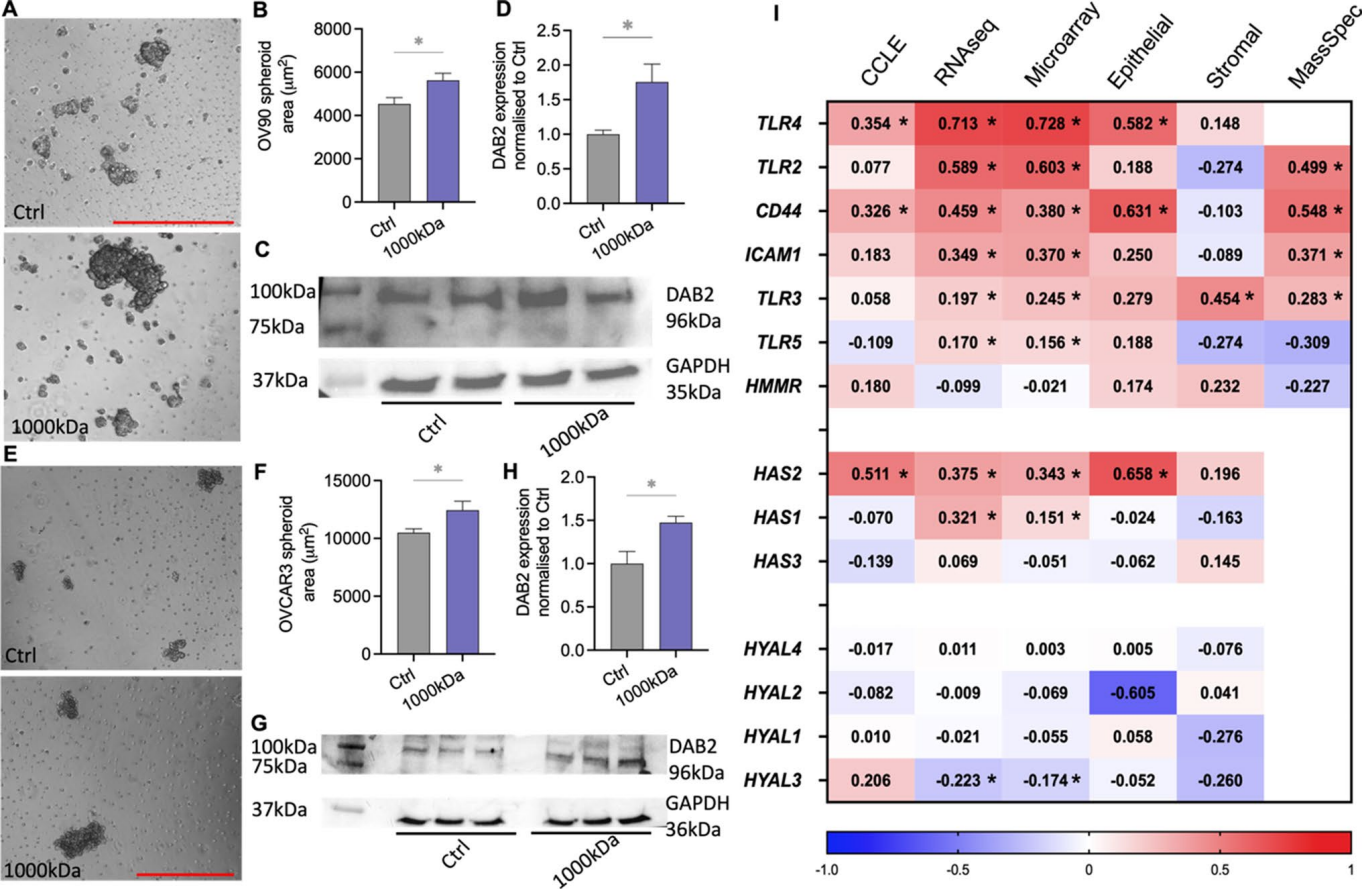
Effects of 1000 kDa HA on HGSOC spheroids **A** Representative images of OV90 spheroids treated with control (Ctrl) or 1000 kDa HA (50 µg/mL) for 72 h. Scale bar 500 µm. **B** Quantitation of OV90 spheroids area treated with Ctrl or 1000 kDa HA (50 µg/mL) (n = 6). **C** Western blot analysis of DAB2 protein expression in OV90 spheroids treated with Ctrl or 1000 kDa HA for 72 h. **D** Quantitation of DAB2 protein expression normalized to Ctrl (n = 5). **E** Representative images of OVCAR3 spheroids treated with Ctrl or 1000 kDa HA (50 µg/mL) for 72 h. Scale bar 500 µm. **F** Quantitation of OVCAR3 spheroids area treated with Ctrl or 1000 kDa HA (n = 3). **G** Western blot analysis of DAB2 protein expression in OVCAR3 spheroids treated with Ctrl or 1000 kDa HA for 72 h. **H** Quantitation of DAB2 protein expression normalized to Ctrl (n = 3). **I** Heat map of Spearman’s rank correlation coefficients for *DAB2* and HA synthesis and degradation enzymes, receptors and co-receptors. Data presented from the TCGA Firehouse dataset [RNA sequencing (n = 307), microarray (n = 558), mass spectrometry (n = 174)], CCLE dataset (n = 64, ovarian adenocarcinoma cell lines) and GSE40595 dataset (epithelium and stroma, n = 32 HGSOC patients). Values = R coefficients, *p < 0.05. All data presented as mean ± SD. *p < 0.05 unpaired t test

We further examined the relationship of DAB2 and 1000 kDa HA in two HGSOC cell lines with moderate (OV90) and high (OVCAR3) Notch3 expression (Supplementary Fig. S2A-B). 1000 kDa HA significantly enhanced spheroid formation by OV90 cells compared to control (Fig. 2A, B, p = 0.031). DAB2 expression was significantly increased in 1000 kDa HA treated OV90 spheroids compared to control (Fig. 2C, D, fold change 1.7, p = 0.035). 1000 kDa HA significantly enhanced spheroid formation compared to control (Fig. 2E, F, p = 0.044) by OVCAR3 cells. DAB2 expression was significantly increased in 1000 kDa HA treated OVCAR3 spheroids compared to control (Fig. 2G, H, fold change 1.47, *p = 0.04). We utilised online databases, GENT2 and cBioportal (TCGA and Broad Institute CCLE datasets), to assess the correlation between *DAB2* and HA related genes. *DAB2* expression had a significant positive correlation with HA synthesis protein *HAS2*, HA receptor *CD44* and co-receptor toll like receptor 4 (*TLR4*) in both ovarian cancer cell lines (CCLE) and patient tissues (TCGA: microarray and RNAsequencing data) (Fig. 2I). Analysis of dataset GSE40595 showed this relationship prelevant to the ovarian cancer epithelium (Fig. 2I). Additionally, *DAB2* showed a significant positive correlation with co-receptors *TLR2,3,5* and *ICAM1*, HA synthesis protein (*HAS1*) and a negative correlation with HA degradation enzyme (*HYAL3*) in the TCGA dataset (Fig. 2I, RNAseq, Microarray). The positive correlation between DAB2 protein and CD44, ICAM1 and TLR2-3 was also significant at the protein level (Fig. 2I, Mass Spec). We went on to further explore the role of DAB2 in ovarian cancer.

### *DAB2* is downregulated in primary ovarian cancer but upregulated in metastatic ovarian cancer

**Fig. 3:**
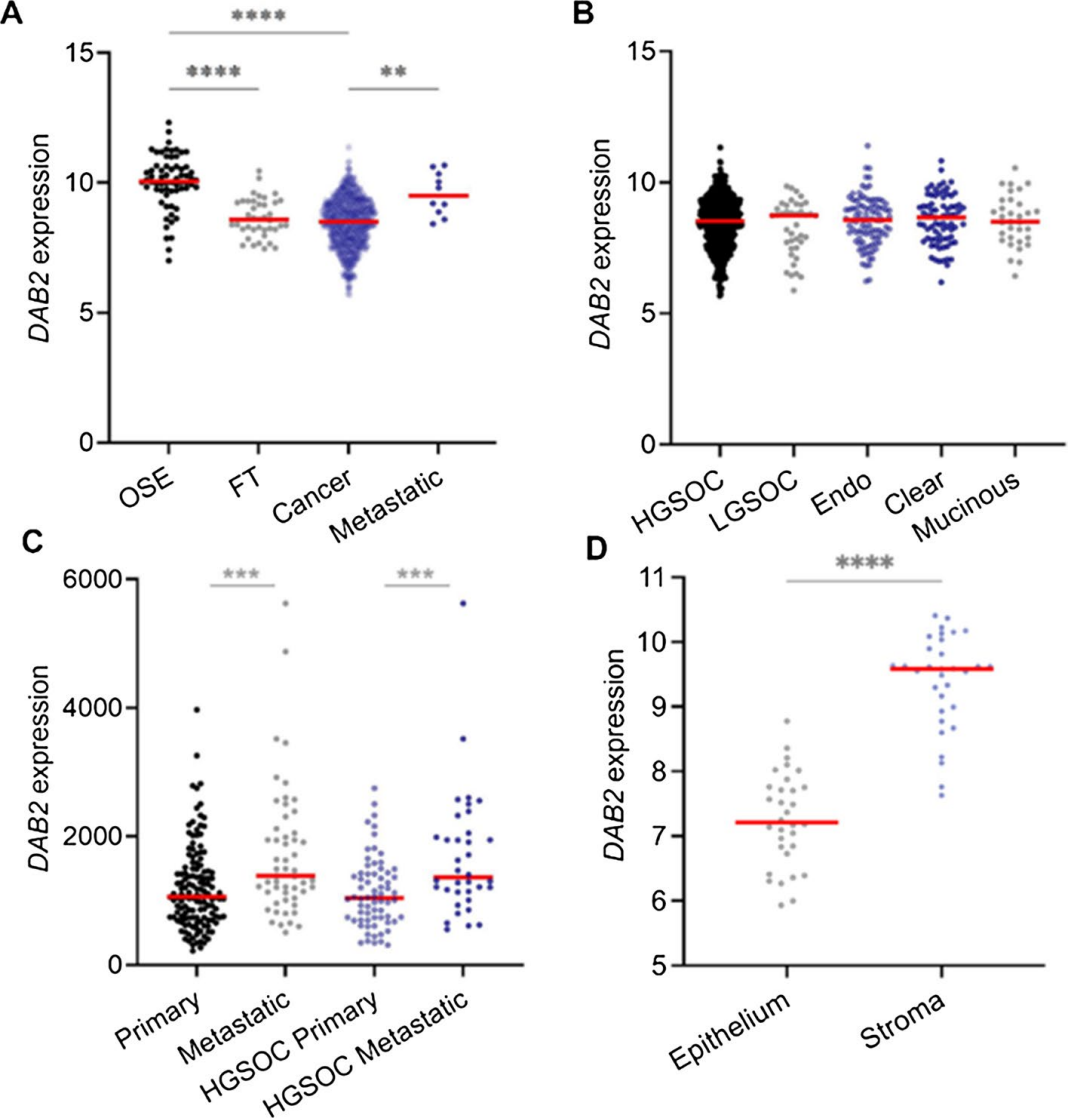
*DAB2* expression in ovarian cancer tissues. **A** *DAB2* expression (GENT2) in OSE (n = 66), FT (n = 40), all epithelial ovarian cancer subtypes (n = 1122) and metastatic ovarian cancer (n = 10). **B** *DAB2* expression (GENT2) in HGSOC (n = 807), LGSOC (n = 40), endometrioid (endo, n = 98), clear cell (n = 77) and mucinous ovarian cancer (n = 33). **C** *DAB2* expression (GSE2109) in primary (n = 138) and metastatic (n = 53) ovarian cancer and primary HGSOC (n = 68) and metastatic HGSOC (n = 36). **D** *DAB2* expression (GSE40595) in laser microdissected epithelium and stroma of matching HGSOC tissues (n = 28). Statistical tests include Kruskal Wallis Dunn’s Multiple Comparison Test (**A**, **B**), Mann Whitney test (**C**) and Wilcoxon test (**D**); ****p < 0.0001, ***p < 0.001, **p < 0.01. Data presented as mean ± SD

Using the GENT2 database, we analysed *DAB2* expression in normal tissues (OSE and FT) and ovarian cancer tissues. *DAB2* expression was significantly decreased in ovarian cancer and FT compared to OSE (Fig. 3A, ****p < 0.0001). Interestingly, *DAB2* expression was significantly increased in metastatic ovarian cancer compared to primary ovarian cancer tissue (Fig. 3A, **p = 0.0055). There were no significant differences in *DAB2* expression between the different ovarian cancer subtypes (Fig. 3B). In another independent dataset (GSE2109), *DAB2* expression was significantly increased in metastatic ovarian cancer tissues compared to primary ovarian cancer tissues for all ovarian cancer subtypes (Fig. 3C, ***p = 0.0009) and for HGSOC tissues (Fig. 3C, ***p = 0.0001). Laser microdissected stromal and epithelium primary HGSOC tissues (GSE40595) showed significantly higher stromal *DAB2* expression compared to the epithelium *DAB2* expression (Fig. 3D, ****p < 0.0001).

### High DAB2 is associated with poor prognosis in ovarian cancer patients

**Fig. 4:**
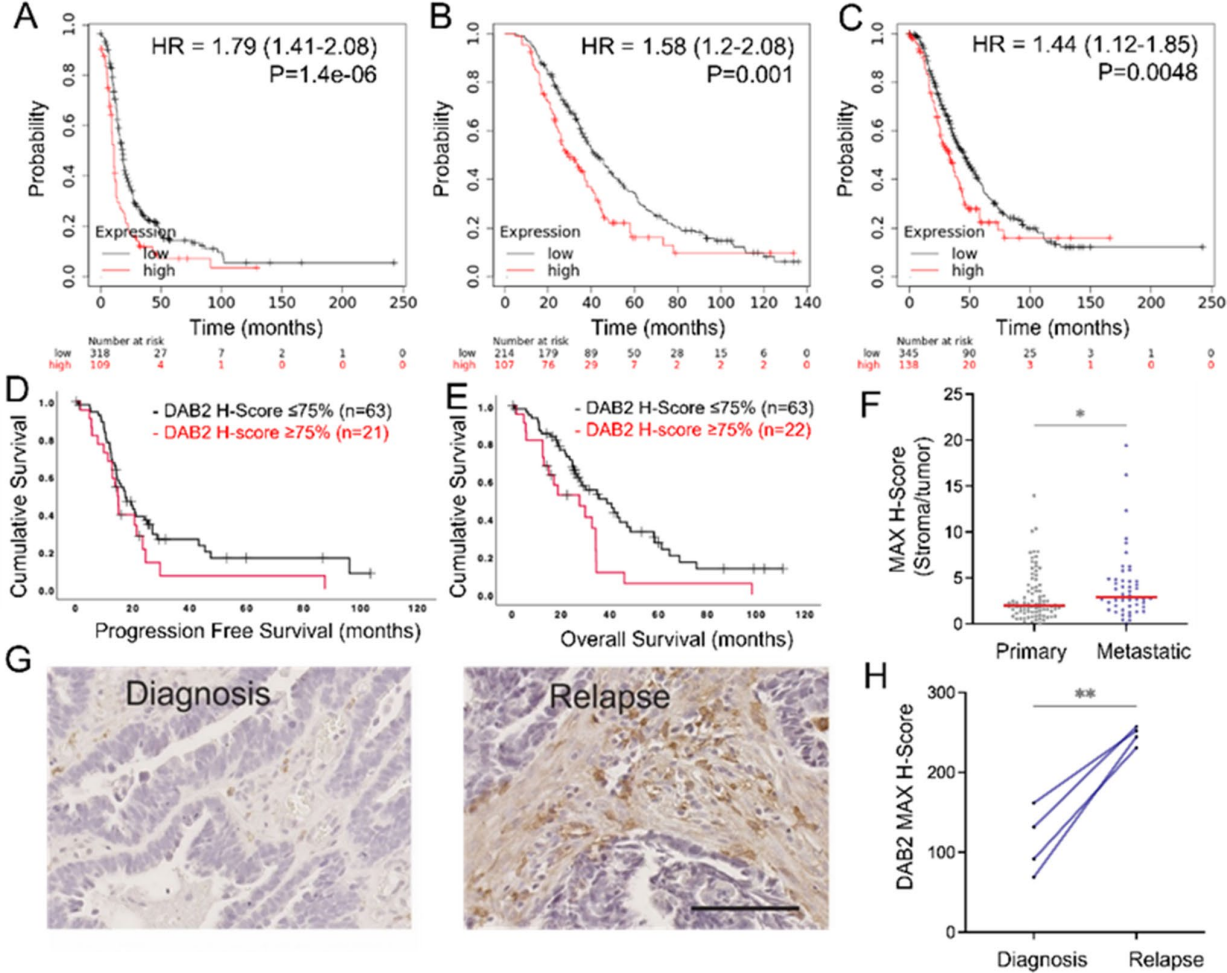
High *DAB2* expression is associated with poor prognosis in HGSOC patients. Kaplan Meier survival plots showing association of *DAB2* expression with **A** PFS (n = 427), **B** PPS (n = 321) and **C** OS (n = 483) in HGSOC patients. TMA slides were immunostained for DAB2 (Abcam, ab256524, 1/800). Kaplan–Meier survival plots for stromal DAB2 H-score in HGSOC tissues for **D** PFS (n = 84, p = 0.122) and **E** OS (n = 85, p = 0.006). **F** The ratio of stromal DAB2 H-score over tumor H-score in primary (n = 87) and metastatic (n = 48) HGSOC TMA tissues (Mann–Whitney U test, n = 48–87, *p = 0.019). Data presented as mean ± SD. **G** Representative images of stromal DAB2 immunostaining in matched patient tissues at diagnosis and relapse (scale bar 50 μM). **H** H-score DAB2 staining in tumor associated stroma in matched HGSOC patient tissues at diagnosis and relapse (n = 4, Paired T test, **p = 0.0049)

DAB2 is an established tumor suppressor, particularly in ovarian cancer [49, 75]. We saw significant increases in *DAB2* in metastatic tissues and therefore examined the relationship between DAB2 mRNA and protein expression and HGSOC patient prognosis (Fig. 3). Online database Kaplan Meier plotter showed high *DAB2* expression was significantly associated with reduced PFS (Fig. 4A, HR = 1.79; 95% CI 1.41–2.28, p = 1.4e−06), PPS (Fig. 4B, HR 1.58; 95% CI 1.2–2.08, p = 0.001) and OS (Fig. 4C, HR: 1.44; 95% CI 1.12–1.85, p = 0.0048) [30]. In an independent HGSOC TMA cohort, patients with highest DAB2 (Q4, H-score ≥ 125) had significantly reduced OS (Fig. 4E; p = 0.006) but not PFS (Fig. 4D; p = 0.122). Epithelial DAB2 was not significantly associated with HGSOC patient prognosis (Supplementary Fig. S5A-B). The ratio of stromal to tumor DAB2 staining was significantly increased in metastatic compared to primary HGSOC tissue (Fig. 4F, p = 0.019). Stromal DAB2 immunostaining was significantly increased in relapse tissues compared to matched tissues at diagnosis (Fig. 4G, H, p = 0.0049, n = 4). No significant difference in epithelial DAB2 expression between tissues at relapse and diagnosis was observed (Supplementary Fig. S5C).

### *DAB2* expression positively correlates with epithelial to mesenchymal transition (EMT) markers in ovarian cancer cell lines and tissues

**Fig. 5:**
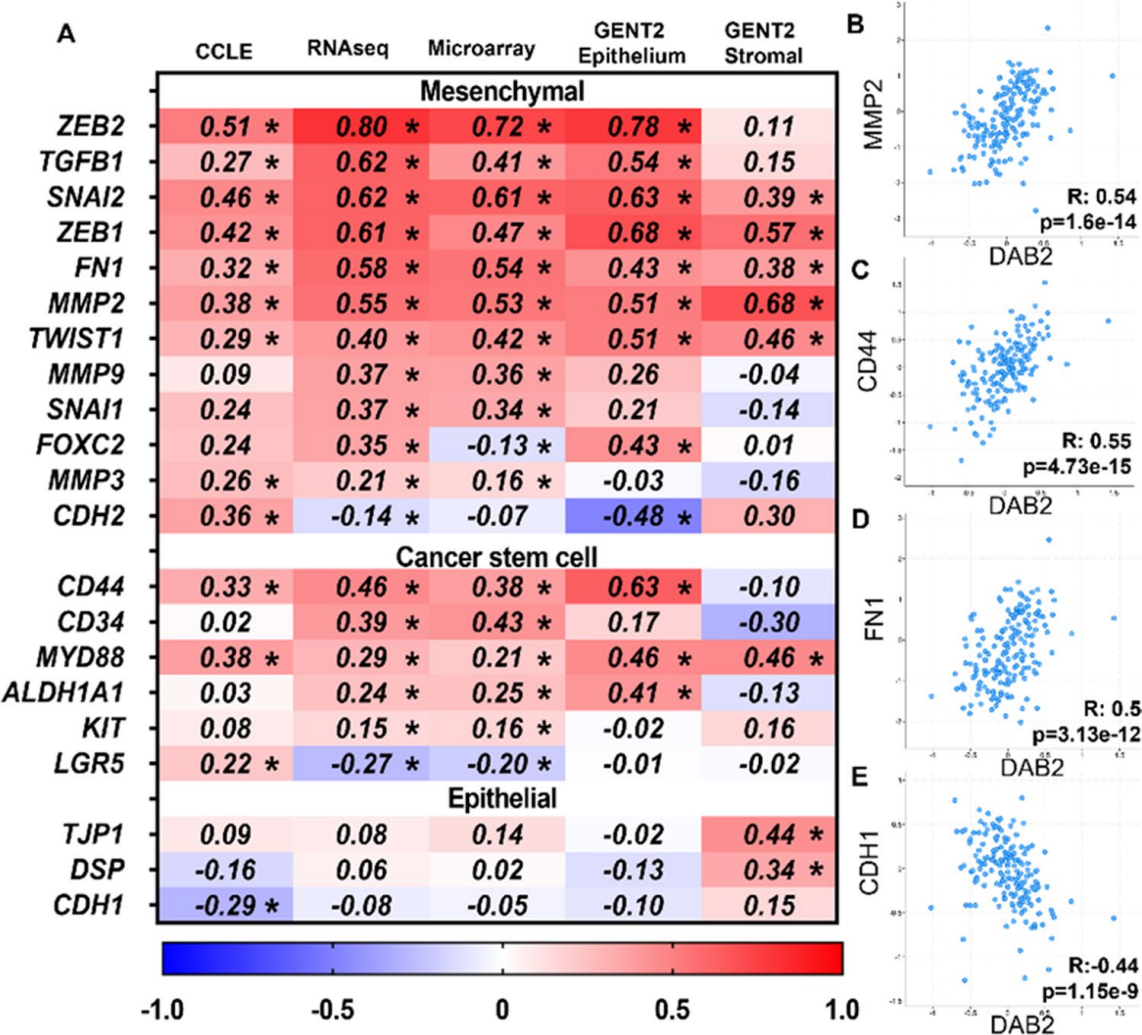
*DAB2* expression correlates EMT and CSC markers in ovarian cancer **A** Heat map of Spearman’s rank correlation coefficients for *DAB2* and mesenchymal, CSC and epithelial markers. Data presented is from cBioPortal analysis of the TCGA Firehouse dataset [RNA sequencing (n = 307) and microarray (n = 558)] and CCLE dataset (n = 64, ovarian adenocarcinoma cell lines) and GENT2 analysis of GSE40595 dataset (n = 32, HGSOC epithelium and stroma). Values = R coefficients, *p < 0.05. cBioPortal spearman correlation analysis of DAB2 expression with **B** MMP2 (R: 0.54, p = 1.6e−14), **C** CD44 (R: 0.55, p = 4.73e−15), **D** FN1 (R: 0.5, p = 3.13e−12) and **E** CDH1 (R: − 0.44, p = 1.15e−9) (TCGA: CPTAC mass spectrometry data, n = 174, TCGA)

To further investigate the role of DAB2 in ovarian cancer progression, we utilized online datasets to assess the relationship of DAB2 expression with EMT markers and CSC markers in ovarian cancer cell lines and tissues. *DAB2* expression had significant positive correlations with mesenchymal markers (*ZEB2, TGFβ1, SNAI2, ZEB1, FN1, MMP2, TWIST1* and *MMP3)* and CSC markers (*CD44* and *MYD88)* in ovarian cancer cell lines (CCLE) and tissues (TCGA: RNA sequencing and microarray) (Fig. 5A). In the ovarian cancer tissues, further significant positive correlations were observed between *DAB2* and CSC markers (*CD34, ALDH1A1, CD117* (Kit)) and mesenchymal markers (*MMP9, SNAI1* and *MMP3)* and negative correlations with mesenchymal markers (*CDH2, FOXC2* and *SOX10*) (Fig. 5A). At a protein level, there was a significant positive correlation between DAB2 and mesenchymal markers MMP2 (Fig. 5B), CD44 (Fig. 5C) and FN1 (Fig. 5D) and a significant negative correlation with epithelial marker CDH1 (Fig. 5E). Overall, this data supports a positive relationship between DAB2 EMT and CSC markers.

### DAB2 is tumor suppressive and promotes therapy resistance in OVCAR3 cells

**Fig. 6:**
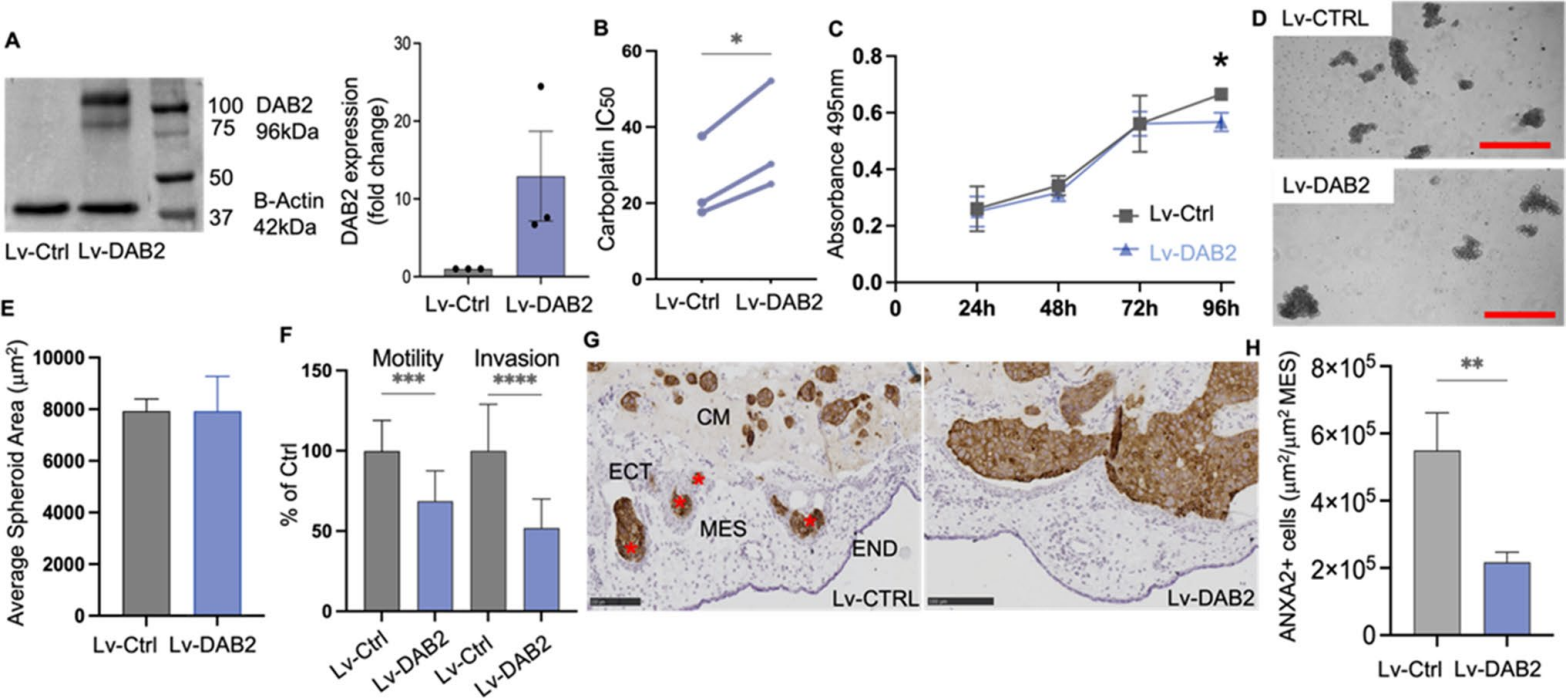
DAB2 has tumor suppressive functions and promotes chemotherapy resistance in OVCAR3 cells. **A** Western blot confirming overexpression of DAB2 in OVCAR3-Lv-DAB2 cells. **B** Carboplatin IC_50_ (μM) dose response in OVCAR3-Lv-Ctrl and OVCAR3-Lv-DAB2 cells (n = 3 experiments, paired-T test, *p < 0.05). **C** OVCAR3-Lv-Ctrl and OVCAR3-Lv-DAB2 cell metabolism at 24 h, 48 h, 72 h and 96 h (n = 3 experiments, n = 12, Šídák's multiple comparisons test, *p < 0.05). **D** Representative images of OVCAR3-Lv-Ctrl and OVCAR3-Lv-DAB2 spheroids cultured at 120 h (scale bar 500 μM). **E** Quantitation of OVCAR3-Lv-Ctrl and OVCAR3-Lv-DAB2 spheroid size (n = 3 experiments, n = 9). **F** Motility and invasion of OVCAR3-Lv-Ctrl and OVCAR3-Lv-DAB2 cells in ChemoTx^®^ invasion assays (n = 3 experiments, n = 18–22, unpaired t-test, ***p < 0.001, ****p < 0.0001). **G** Representative images showing invasion of OVCAR3-Lv-Ctrl and OVCAR3-Lv-DAB2 cells (ANXA2 positive immunostaining, labelled with red asterisks) from the Matrigel into the mesoderm of the CAM. *CM *cancer cells in matrigel implant, *ECT *ectoderm, *MES *mesoderm, *END *endoderm. Scale bar 100 µm. **H** Quantitation of invaded OVCAR3 cells presented as area of positive cells μm^2^/area of mesoderm mm^2^ (n = 2 experiments, n = 11–13 chicken embryos, Unpaired t-test, **P < 0.0053). Data presented as mean ± SD

OVCAR3 and A2780 cells were selected for functional assays as both cell lines have low DAB2 mRNA and protein expression (Supplementary Fig. S2C-D). Using lentiviral plasmids, we overexpressed DAB2 in OVCAR3 cells and confirmed DAB2 overexpression (OVCAR3-Lv-DAB2 cells) by Western blot (Fig. 6A, mean fold change 12.9). DAB2 significantly enhanced the IC_50_ dose to carboplatin in OVCAR3-Lv-DAB2 cells compared to OVCAR3-Lv-Ctrl cells (Fig. 6B). MTT assays showed a small significant reduction in cell metabolism at 96 h in OVCAR3-Lv-DAB2 cells in comparison to OVCAR3-Lv-Ctrl cells (Fig. 6C, p = 0.018). DAB2 had no effect on spheroid formation of OVCAR3 cells (Fig. 6D, E). Overexpression of DAB2 (OVCAR3-Lv-DAB2 cells) significantly reduced cell motility and invasion in vitro compared to OVCAR3-Lv-Ctrl cells (Fig. 6F, motility p = 0.003, invasion: p < 0.0001). OVCAR3-Lv-DAB2 cells had significantly reduced invasion in vivo into the endoderm and mesoderm compared to OVCAR3-Lv-Ctrl cells (Fig. 6G, H, p = 0.0053).

### DAB2 has both tumor suppressive and tumor promoting functions in A2780 cells

**Fig. 7:**
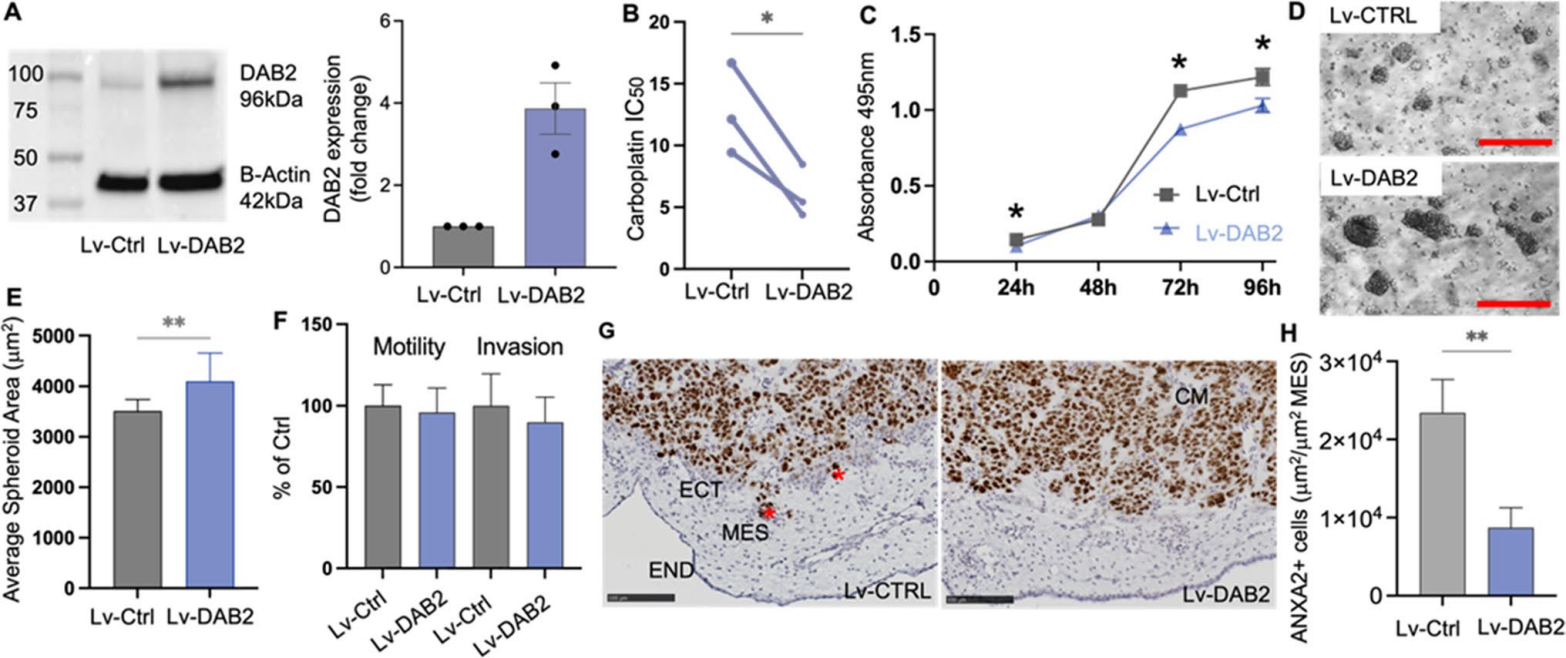
DAB2 has tumor suppressive and tumor promoting functions in A2780 cells **A** Western blot analysis confirming lentiviral overexpression of DAB2 expression in A2780 cells (n = 4). **B** Carboplatin IC_50_ (μM) dose response in A2780-Lv-Ctrl and A2780-Lv-DAB2 cells (n = 3 experiments, paired-T test, *P < 0.05). **C** A2780 Lv-Ctrl and Lv-DAB2 cell metabolism at 24 h, 48 h, 72 h and 96 h (3 experiments, n = 12, 2-way ANOVA, Šídák’s multiple comparisons test, *p < 0.05). **D** Representative images of A2780-Lv-Ctrl and A2780-Lv-DAB2 spheroids at 72 h. Scale bar 250 µM. **E** Quantitation of A2780-Lv-Ctrl and A2780-Lv-DAB2 spheroid size (3 experiments, n = 9, unpaired t-test, **P = 0.0097). **F** Motility and invasion of A2780-Lv-Ctrl and A2780-Lv-DAB2 cells in the ChemoTx^®^ system in vitro (n = 3 experiments, n = 18–22, unpaired t-test). **G** Representative images showing invasion of A2780-Lv-Ctrl and A2780-Lv-DAB2 cells (Ki67 positive immunostaining, labelled with red asterisks) from matrigel into the ectoderm and mesoderm of the CAM. *CM *cancer cells in matrigel implant, *ECT *ectoderm, *MES *mesoderm, *END *endoderm. Scale bar 100 µm. **H** Quantitation of A2780 invaded cells presented as area of Ki67 positive cells μm^2^/area of mesoderm mm^2^ (n = 2 experiments, n = 13–14 chicken embryos, Unpaired t-test, **P < 0.0053). Data presented as mean ± SD

We overexpressed DAB2 in A2780 cells using lentiviral systems and confirmed DAB2 overexpression by Western blot (Fig. 7A, mean fold change 3.9). The carboplatin IC_50_ of A2780-Lv-DAB2 cells was significantly decreased compared to A2780-Lv-Ctrl (Fig. 7B, p = 0.0378), suggesting DAB2 enhances the sensitivity of A2780 cells to carboplatin. A2780-Lv-DAB2 cells had significantly reduced cell metabolism at 24 h, 72 h and 96 h compared to A2780-Lv-Ctrl cells (Fig. 7C). No difference in cell survival was observed at 48 h. Interestingly, A2780-Lv-DAB2 cells had significantly enhanced spheroid formation compared to A2780-Lv-Ctrl cells (Fig. 7D, E, 72 h, p = 0.0097). There were no significant effects of DAB2 overexpression on A2780 cell motility and invasion in vitro (Fig. 7F). However, similar to OVCAR3 cells, A2780-Lv-DAB2 cells had significantly reduced cell invasion in vivo in the CAM model compared to A2780-Lv-Ctrl cells (Fig. 7G, H, p = 0.0059).

### *DAB2* expression is enhanced in pro-tumorigenic M2-macrophages

**Fig. 8:**
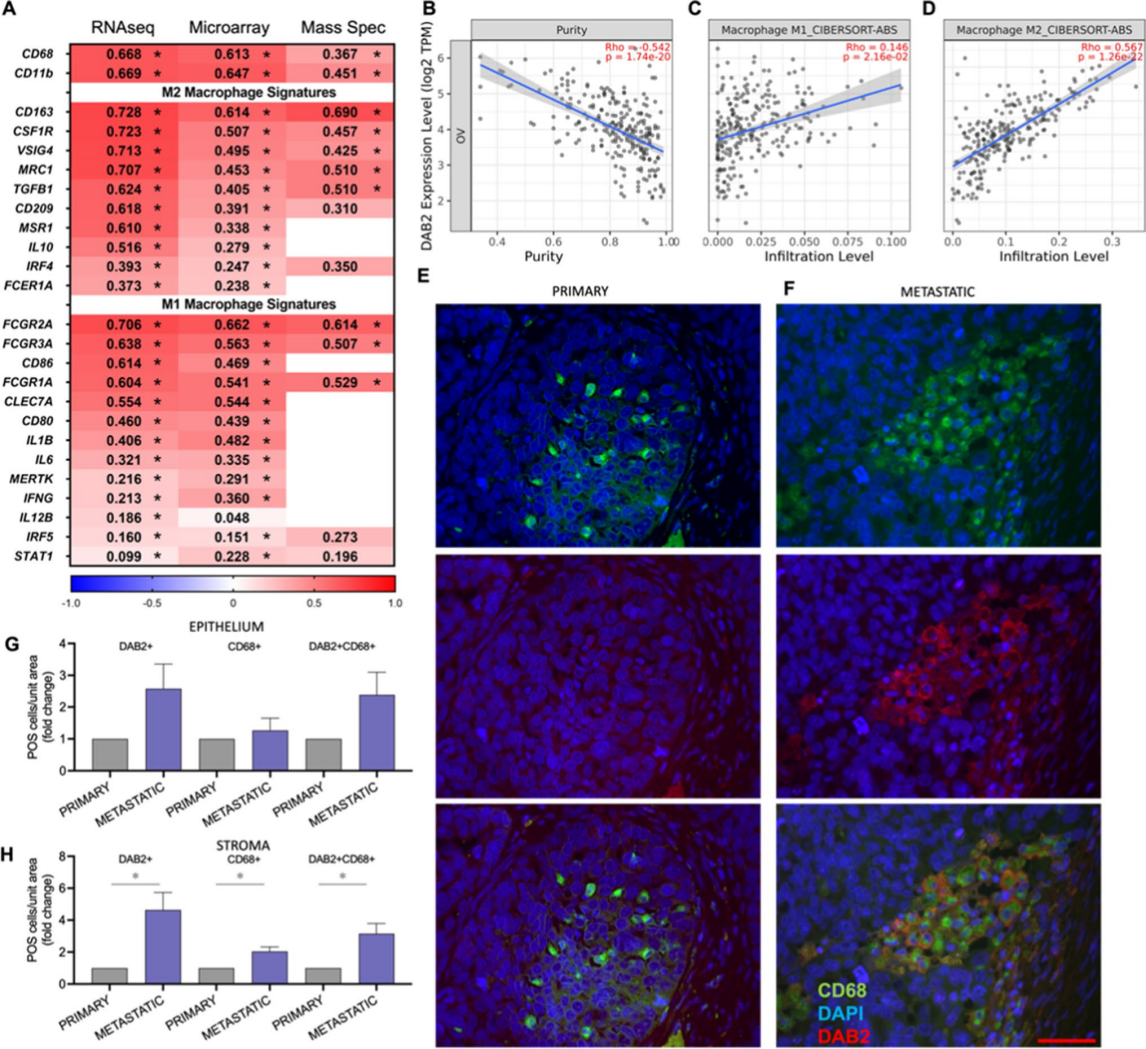
Relationship between DAB2 and tumor associated macrophages (TAMs) in ovarian cancer patient tissues. **A** Heat map of Spearman’s rank correlation coefficients for the relationship between DAB2 and general macrophage, M1 polarized macrophage and M2 polarized macrophage signatures. Data presented is from cBioPortal analysis of TCGA Firehouse dataset (RNA sequencing (n = 307), microarray (n = 558) and mass spectrometry (n = 174)). Significant coefficients are labelled *p < 0.05. TIMER dataset analysis of relationship between DAB2 (log_2_ transcripts per million (TPM)) and **B** tumor cell purity, **C** M1 macrophages and **D** M2 macrophages in TCGA data from ovarian cancer patient tissues. Immunofluorescence visualization of DAB2 (1/100, Abcam, ab256524, red) and CD68 (1/400, Abcam, ab955, green) in matched HGSOC patient tissue from **E** primary and **F** metastatic disease (scale bar 50 µm). Quantitation of DAB2+, CD68+ and DAB2+ CD68+ cells normalized to area for tumor epithelium **G** and tumor associated stroma **H** in matched HGSOC patient tissue in primary and metastatic disease presented as fold change in metastatic compared to matched primary tissue (n = 5 matched patient tissues)

cBioPortal co-expression analysis showed strong significant positive correlations between DAB2 expression and multiple signatures and markers for general macrophages, M1-polarized macrophages and M2-polarized macrophage (Fig. 8A). The relationship between *DAB2* expression and macrophages was confirmed in the TIMER dataset, which examines immune infiltrates in ovarian cancer tissues in the TCGA dataset. In their modelling system, *DAB2* was negatively correlated with tumor purity indicating a stronger relationship with cells in the tumor microenvironment (Fig. 8B, R = − 0.542, p = 1.74e^−20^). There was a significant relationship with M1-polarized macrophages (Fig. 8C, Rho = 0.146, p = 2.16e^−2^) but a stronger relationship with M2-polarized macrophages (Fig. 8D, Rho = 0.567, p = 1.26e^−22^). Using immunofluorescence, we found DAB2 to co-localize with CD68 macrophage marker in both the cancer associated stroma and within epithelial areas of HGSOC tissue (Fig. 8E, F). We assessed the proportion of DAB2+, CD68+ and double labelled (DAB2+ CD68+) cells in matched HGSOC patient tissues from primary and metastatic cancers (Fig. 8E, F). There was no significant difference between DAB2+, CD68+ or DAB2+ CD68+ cell populations in tumor epithelium (Fig. 8G). In the tumor associated stroma, the proportion of DAB2+ (fold change 4.64, p = 0.029), CD68+ (fold change 2.04, p = 0.02) and DAB2+ CD68+ (fold change 3.15, p = 0.028) was significantly increased in metastatic compared to matched primary tumors (Fig. 8H).

## Discussion

There is increasing literature on the pro-tumorigenic functions of HA signaling in cancer. However, there is limited knowledge on how molecular weight of HA impacts its function. In ovarian cancer, relapse of chemotherapy resistant disease is common and contributes strongly to its poor survival rates [37]. Investigation of different molecular weight HA in ovarian cancer provides the opportunity to identify novel targets for therapy. This study identified DAB2 as a novel protein regulated by 1000 kDa HA in ovarian cancer. *DAB2* was downregulated in ovarian cancer compared to normal OSE and high *DAB2* expression was associated with poor patient outcome. Interestingly, *DAB2* was increased in metastatic ovarian cancer tissues compared to primary disease and positively associated with EMT and CSC markers. Stromal DAB2 was associated with reduced OS and relapse. DAB2 overexpression had both tumor suppressive and promoting functions in A2780 and OVCAR3 cells, however DAB2 overexpression strongly inhibited their in vivo invasion in the chicken CAM assay. *DAB2* was positively correlated with M1 and M2 macrophages in ovarian cancer tissues and there was a significant increase in DAB2 positive macrophages in tumor associated stroma of metastatic ovarian cancer compared to matched primary tissues.

HA activates CSC signaling pathways and enhances spheroid formation in cancers of the breast, head and neck and ovary [5, 6, 37]. In breast cancer, 35 kDa HA and not 117 kDa HA enhanced spheroid formation of 4 T-1 and SKBR3 breast cancer cells [79]. We previously found inhibiting HA production by 4-MU treatment significantly decreased spheroid formation of OV90 ovarian cancer cells and chemoresistant patient derived cells [37]. A study by Shiina et al*.* showed 200 kDa HA enhanced the expression of pluripotency genes (*NANOG, SOX2, POU5F1* and *KLF-4)* in HSC-3 cells selected for CSC markers ALDH and CD44v3 [60]. However, 5 kDa, 20 kDa and 700 kDa HA had no significant effects [60]. Based on these findings this study sought to identify novel targets of HA signaling by investigating effects of different molecular weight HA in ovarian cancer stem like populations. Notch3 signaling has been shown to promote the existence of CSC populations and stem-like features including spheroid formation and therapy resistance [45]. Inhibition of Notch3 decreased the proportions of ALDH1A1 and CD44 positive cells in A549 and H520 non-small lung cancer cells [39]. In OVCAR3 HGSOC cell line, Notch3 knockdown sensitised cells to chemotherapy [53]. Another ovarian cancer study demonstrated that side population cells with enhanced CSC features had high Notch3 expression and inhibition of Notch signaling increased response to chemotherapy and reduced tumor burden in vivo [45]. In this study we overexpressed the active part of Notch3, NICD3, in ES-2 cells and observed significantly enhanced spheroid formation. This effect on spheroid size was further increased when ES-2-Rv-NICD3 were combined with WT ES-2 cells (3:1) which we hypothesise could be due to intercellular signaling as ES-2 highly express Notch co-activator ligand Jagged-1, shown to regulate Notch3 signaling in ovarian cancer [9].

Our findings showed that HA molecular weight impacted spheroid formation, 1000 kDa HA significantly enhanced spheroid formation, compared to control, 27 kDa and 183 kDa HA, in ES-2 (ES-2:ES-2-Rv-NICD3, 1:3) cells. Mass spectrometry identified DAB2 as a novel protein upregulated by 1000 kDa HA signaling in ES-2:ES-2-Rv-NICD3 spheroids. We further validated 1000 kDa HA enhanced spheroid formation and DAB2 expression in two HGSOC cell lines with moderate to high Notch3 expression (OVCAR3 and OV90). To the best of our knowledge, this is the first study to demonstrate DAB2 expression is regulated by HA. Interestingly, in ES-2 WT cells, 1000 kDa HA had no effects on spheroid formation or DAB2 expression. Inhibition of HA synthesis by 4-MU reduced DAB2 expression in ES-2:ES-2-Rv-NICD3 but not ES-2-WT cells. Together these results suggest the effect of HA on DAB2 expression in ovarian cancer may be dependent on Notch3. There is limited literature on the relationship between Notch3 and HA, although Notch3 has been shown to promote expression of CSC marker and HA receptor CD44 in small lung cancer [39]. Additionally, sulfated HA has been shown to enhance expression of Notch3 in keratinocytes [50]. Further experiments are required to determine if Notch3 inhibition can block the HA effect on DAB2 expression.

DAB2 was initially identified as a novel cDNA fragment downregulated in ovarian cancer cell lines compared to normal ovarian cell lines [48]. Downregulation of DAB2 expression has been observed in cancers of the breast, placenta, lung, esophagus, cervix, stomach, prostate and nasopharynx [8, 17, 29, 43, 66, 68, 72, 75, 77]. In this study, we reported *DAB2* expression was significantly reduced in ovarian cancer compared to normal OSE, consistent with previous ovarian cancer studies [14, 49, 75]. No significant differences in *DAB2* expression in ovarian cancer subtypes was observed. Interestingly, Mok et al*.* found DAB2 staining was maintained in all mucinous ovarian tumors irrelevant of grade, whilst 20% of other grade I tumors had detectable DAB2 staining with no DAB2 detected in grade II or III tumors [49]. These differences may be due quantitation of stromal or epithelial tumor areas or antibody clonal differences and methods used for immunostaining. Previous studies demonstrated that low DAB2 protein expression is associated with poor patient prognosis in patients with cancers of the lung, bladder and esophagus [11, 28, 68, 72]. However, our analysis found stromal but not epithelial DAB2 expression was associated with poor outcome in HGSOC patients. This is consistent with a study in urothelial carcinoma of the bladder, where stromal DAB2 but not epithelial DAB2 was associated with reduced PFS [26]. Online microarray data for HGSOC patients, including both stroma and epithelium, showed high *DAB2* expression was associated with reduced PFS, PPS and OS. Together these findings suggest that the pro-tumorigenic roles of DAB2 may be mediated by cells in the tumour–associated stroma.

EMT is a key mechanism in tumor metastasis [25]. The role of DAB2 in EMT is very conflicting, with evidence supporting both activation [7, 26, 56, 68] and inhibition [22, 43, 78]. We observed strong positive correlations between DAB2 and EMT markers in online database analysis of ovarian cancer cell lines and patient tissues. The principal function of DAB2 is an endocytic adaptor protein in clathrin mediated endocytosis [55]. DAB2 has key binding domains and motifs to recognize and recruit proteins to clathrin coated pits. Two key regions include the phosphotyrosine binding (PTB) domain and proline rich domain (PRD). DAB2 PRD interacts with Src homology domain 3 (SH3) domain of growth factor receptor bound protein 2 (Grb2) preventing its binding to son of sevenless (SOS), inhibiting canonical MAPK activation [80]. Loss of DAB2 was associated with MAPK activation and enhanced cell proliferation, migration and therapy resistance in C4-2 prostate cancer cells [80]. DAB2 also directly interacts with the TGF*β* pathway with the DAB2 PTB domain binding both Smad2 and Smad3 [23, 55]. Downregulation of DAB2 significantly increases the association of SOS with Grb2 in M1 breast cancer cells, enhancing ERK phosphorylation and activating TGFβ signaling mediated EMT [43]. DAB2 knockdown in pancreatic cell lines also activated MAPK and subsequent expression of EMT markers (Snail and Slug), further enhanced by TGFβ stimulation [22]. This relationship between MAPK and TGFβ signaling was more complex in esophageal small cell carcinoma (ESCC). DAB2 inhibition significantly activated the MAPK pathway and enhanced wound healing, cell migration and colony formation in ESCC cells [68]. In KYSE-50 ESCC cells with high DAB2, stimulation with TGFβ1 promoted EMT, through enhanced N-cadherin and decreased E-cadherin expression [68]. Furthermore, this increase in N-cadherin and decrease in E-cadherin expression was associated with reduced survival in ESCC patient tissues with high DAB2 expression [68]. Together, these findings highlight how external factors may be crucial in determining the function of DAB2.

In this study we observed different effects of DAB2 on OVCAR3 and A2780 cells in vitro. A2780 with DAB2 overexpression had enhanced spheroid formation and increased sensitivity to carboplatin, while OVCAR3 had reduced sensitivity to carboplatin but no change in spheroid formation. Cell migration and invasion in vitro was decreased in OVCAR3 cells overexpressing DAB2 but not in A2780 cells. These different effects may be explained by the fact that these cell lines represent different ovarian cancer subtypes. A2780 cells are of endometrioid subtype whilst OVCAR3 are classified as HGSOC [3]. We also hypothesise the difference may be due to presence of different co-activators or proteins related to pathways including TGFβ, which has both tumor suppressive or promoting properties depending on different conditions [55]. Interestingly, DAB2 overexpression significantly reduced cell invasion in the in vivo CAM assay for both A2780 and OVCAR3 cells. In the CAM assay both cell lines were subjected to same microenvironment, suggesting external factors are also important in determining the function of DAB2. Inhibition of cell invasion by DAB2 has also been observed in other studies including oral, hepatocellular, cervical, gastric, lung and esophageal cancers [8, 20, 46, 61, 68, 69, 78]. We found DAB2 overexpression also reduced cell metabolism, indicative of cell survival, in both A2780 and OVCAR3 cells. DAB2 has previously been found to reduce cell proliferation and in vivo tumorigenicity in SKOV3 ovarian cancer cells [7]. This is consistent with other studies in acute myeloid leukaemia, breast cancer, lung, and hepatocellular cancers where DAB2 inhibited cell proliferation [24, 61, 64, 78]. Enhanced proliferation and in vivo tumorigenicity by DAB2 has also been observed in urothelial and prostate cancer highlighting the need for further studies [26, 73].

Previous studies in bladder and lung cancer have found DAB2 expression was downregulated in metastatic tumors compared to primary tumors [28, 72]. Interestingly, our analysis found DAB2 to be enhanced in metastatic compared to primary ovarian cancer tissues, particularly in the stroma. We hypothesize DAB2 in cancer-associated stroma cells may play a pro-tumorigenic role. A study in bladder cancer found secreted factors from DAB2 overexpressing stromal cells promoted EMT in UM-UC-3 cells whilst DAB2 knockdown inhibited EMT [26]. In lung cancer DAB2 downregulation was associated with promoter methylation [72]. Demethylation treatments enhanced DAB2 expression which reduced cell proliferation and migration [72]. Future studies need to assess if demethylation treatments will also be effective at promoting DAB2 expression and reducing ovarian cancer proliferation and migration.

We observed a positive relationship between DAB2 expression and macrophages in ovarian cancer tissues via online expression data and the TIMER dataset. There was a stronger relationship between DAB2 and M2 polarized macrophages. M1 macrophages are tumor-suppressive, driving anti-tumor immune responses whereas M2 macrophages are tumor-promoting and release a range of pro-metastatic secretory factors [41]. In ovarian cancer, macrophages are the most dominant immune cell type with M2-polarized macrophages being the most prevalent macrophage subtype (51%) [12]. Infiltration of M2 macrophages in the metastatic tumor microenvironment in ovarian cancer is associated with significantly reduced OS [21]. DAB2 is associated with polarisation of bone marrow derived macrophages to an M2 phenotype [1]. DAB2 knockdown in tumor associated macrophages (TAMs) has been found to reduce lung metastases of E0771 breast cancer cells in vivo [42]. In this study, we confirmed the co-localization of DAB2 and macrophage marker (CD68) in HGSOC tissue. Furthermore, we found a significant increase in DAB2+, CD68+ and DAB2+ CD68+ cells in tumor associated stroma of metastatic HGSOC compared to matched primary tissues. Our findings suggest that TAMs contribute to the high DAB2 positive cells in the cancer-associated stroma found to be associated with reduced ovarian cancer survival. Further studies including co-culture experiments with TAMs and ovarian cancer cells are required to further scrutinize the role of DAB2 in the tumour microenvironment.

## Conclusions

This is the first study to identify that DAB2 is up-regulated by 1000 kDa HA. Our findings show that DAB2 is associated with ovarian cancer metastasis, HA signaling molecules, EMT and poor prognosis. Consistent with previous literature, DAB2 has inhibitory effects on ovarian cancer cell metabolism, motility and invasion. We demonstrated there was a significant increase in DAB2+ TAMs in metastatic compared to matched primary ovarian cancer tissue that may aid tumour progression. Our findings highlight that DAB2 has a direct tumor suppressive role on ovarian cancer cells. Understanding the pro-tumorigenic roles of DAB2 in the surrounding tumor microenvironment needs further investigation.

### Supplementary Information

Below is the link to the electronic supplementary material.

Supplementary file1 (DOCX 3014 KB)

## Data Availability

The datasets used and/or analysed during the current study are available from the corresponding author on reasonable request.

## Abbreviations

CAM: Chick chorioallantoic membrane
CCLE: Cancer cell line encyclopedia
DAB2: Disabled-2
EMT: Epithelial to mesenchymal
ESCC: Esophageal small cell carcinoma
FT: Fallopian tube
GSE: Genomic spatial events
HA: Hyaluronan
HAS: HA synthase
HGSOC: High grade serous ovarian cancer
HMW-HA: High molecular weight HA
HYAL: Hyaluronidase
LC–MS/MS: Liquid chromatography with tandem mass spectrometry
LGSOC: Low grade serous ovarian cancer
LMW-HA: Low molecular weight HA
NICD3: Notch3 intracellular domain
OS: Overall survival
OSE: Ovarian surface epithelium
PFS: Progression free survival
PPS: Post progression survival
TAM: Tumor associated macrophage
TMA: Tissue microarray
4-MU: 4-Methylumbelliferone
