# Supplementary material for: Disabled-2: a protein up-regulated by high molecular weight hyaluronan has both tumor promoting and tumor suppressor roles in ovarian cancer
Source: Cell Mol Life Sci. 2023 Oct 10;80(11):320. doi: 10.1007/s00018-023-04972-9 (PMC10564841; doi:10.1007/s00018-023-04972-9)
Supplement: Supplementary file 1 — Supplementary file1 (DOCX 3014 KB) [file 18_2023_4972_MOESM1_ESM.docx]

Disabled-2: a protein up-regulated by high molecular weight hyaluronan has both tumor promoting and tumor suppressor roles in ovarian cancer

Cellular and molecular Life Science

Zoe K Price^a,b^, Noor A Lokman^a^, Mai Sugiyama^c,d^, Yoshihiro Koya^c,d^, Masato Yoshihara^b^, Martin K Oehler^a,e^, Hiroaki Kajiyama^b^ and Carmela Ricciardelli^a^*

^a^Discipline of Obstetrics and Gynaecology, Adelaide Medical School, Robinson Research Institute, University of Adelaide, South Australia 5000, Australia

^b^Department of Obstetrics and Gynecology, Nagoya University Graduate School of Medicine, Nagoya, Japan

^c^Bell Research Center, Department of Obstetrics and Gynecology Collaborative Research, Nagoya University Graduate School of Medicine, Nagoya, Japan

^d^Bell Research Center for Reproductive Health and Cancer, Nagoya, Japan

^e^Department of Gynaecological Oncology, Royal Adelaide Hospital, Adelaide 5000, Australia

*Correspondence: Carmela Ricciardelli, Reproductive Cancer Group, AHMS Building, The University of Adelaide, Adelaide, Australia, carmela.ricciardelli@adelaide.edu.au; Tel.:+61-08-8313-8255

**Supplementary Table 1:** TaqMan Primer details

| Gene | Primer ID |
| --- | --- |
| *TWIST1* | Hs00361186_m1 |
| *NOTCH1* | Hs01062014_m1 |
| *NOTCH3* | Hs01128537_m1 |
| *GAPDH* | Hs02758991 |

**Supplementary Table 2:** Clinicopathological characteristics of HGSOC TMA cohort and matching HGSOC patient tissues.

| Primary serous carcinomas (n=87) | | |
| --- | --- | --- |
| Age at Diagnosis (years) | Median (range) | 60 (24-86) |
| Histological Grade | Grade 2 | 18 |
|  | Grade 3 | 69 |
| FIGO stage | Stage II | 1 |
|  | Stage III | 85 |
|  | Stage IV | 1 |
| DAB2ep (n=87), H-score max percentiles | 25 | 20.1 |
|  | 50 | 34.3 |
|  | 75 | 70.4 |
| DAB2st (n=86), H-score max percentiles | 25 | 52.6 |
|  | 50 | 83.6 |
|  | 75 | 125.6 |
| Recurrence | No | 19 |
|  | Yes | 62 |
|  | Unknown | 6 |
| Cause of Death | Ovarian cancer | 59 |
|  | Other cause | 5 |
|  | Alive | 18 |
|  | No follow-up | 1 |
| Metastatic serous carcinomas (n=49) | | |
| Age at Diagnosis (years) | Median (range) | 68 (45-90) |
| Histological Grade | Grade 2 | 8 |
|  | Grade 3 | 41 |
| FIGO stage | Stage III | 44 |
|  | Stage IV | 5 |
| DAB2ep (n=49), H-score max percentiles | 25 | 15.4 |
|  | 50 | 34.3 |
|  | 75 | 60.8 |
| DAB2st (n=48), H-score max percentiles | 25 | 69.4 |
|  | 50 | 100.4 |
|  | 75 | 130.1 |
| Recurrence | No | 14 |
|  | Yes | 30 |
|  | Unknown | 5 |
| Cause of Death | Ovarian cancer | 30 |
|  | Other cause | 3 |
|  | Alive | 14 |
|  | No follow-up | 2 |
| Matched diagnosis and relapse serous carcinomas (n=4) | | |
| Age at Diagnosis | Median (range) | 54 (42-78) |
| Age at relapse | Median (range) | 58 (46-79) |
| FIGO stage | Stage I | 1 |
|  | Stage III | 3 |
| Histological Grade | Grade 3 | 4 |
| Cause of Death | Ovarian cancer | 1 |
|  | Alive | 2 |
|  | No follow-up | 1 |


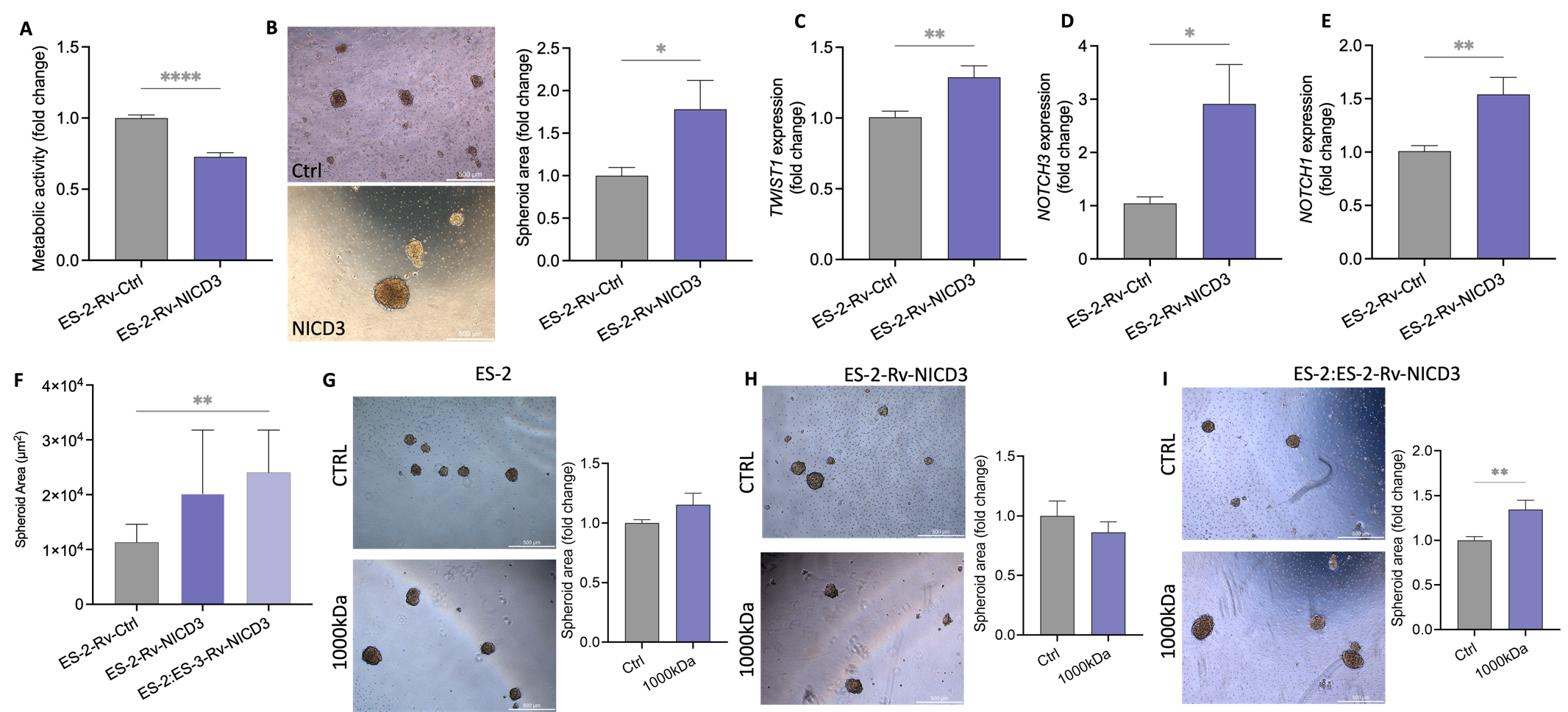


Supplementary Fig. 1. Effects of NICD3 in ES-2 cells. (A) Metabolic activity in ES-2-Rv-Ctrl and ES-2-Rv-NICD3 cells as determined by MTS assay (n=4 experiments, n=16, unpaired t-test, ****p<0.001). (B) Spheroid formation of ES-2-Rv-Ctrl and ES-2-Rv-NICD3 cells (n=3 experiments, n=12, unpaired t test, *p=0.042). Gene expression in ES-2-Rv-NICD3 cells compared to ES-2-Rv-Ctrl of (C) *TWIST1*, (D) *NOTCH3* and (E) *NOTCH1* (n=3 experiments, n=6, unpaired t test, *p<0.05, **p<0.01, gene expression normalised to *GAPDH* using the 2-^∧∧CT^ method). (F) Spheroid area of ES-2-Rv-Ctrl, ES-2-Rv-NICD3 and ES-2:ES-2-Rv-NICD3 (1:3) cells (10,000 cells/well after 72hr culture (n=3 experiments, n=9, Kruskal Wallis test, *p=0.0021). Spheroid formation of (G) ES-2 WT, (H) ES-2-Rv-NICD3 and (I) ES-2:ES-2-Rv-NICD3 (1:3) cells treated with 1000kDa HA (50µg/mL) in 24 well polyHEMA plates after 72hr (10,000 cells/well; n=3 experiments; n=9, **p<0.0012; Mann-Whitney Test). Scale bar = 500µm.


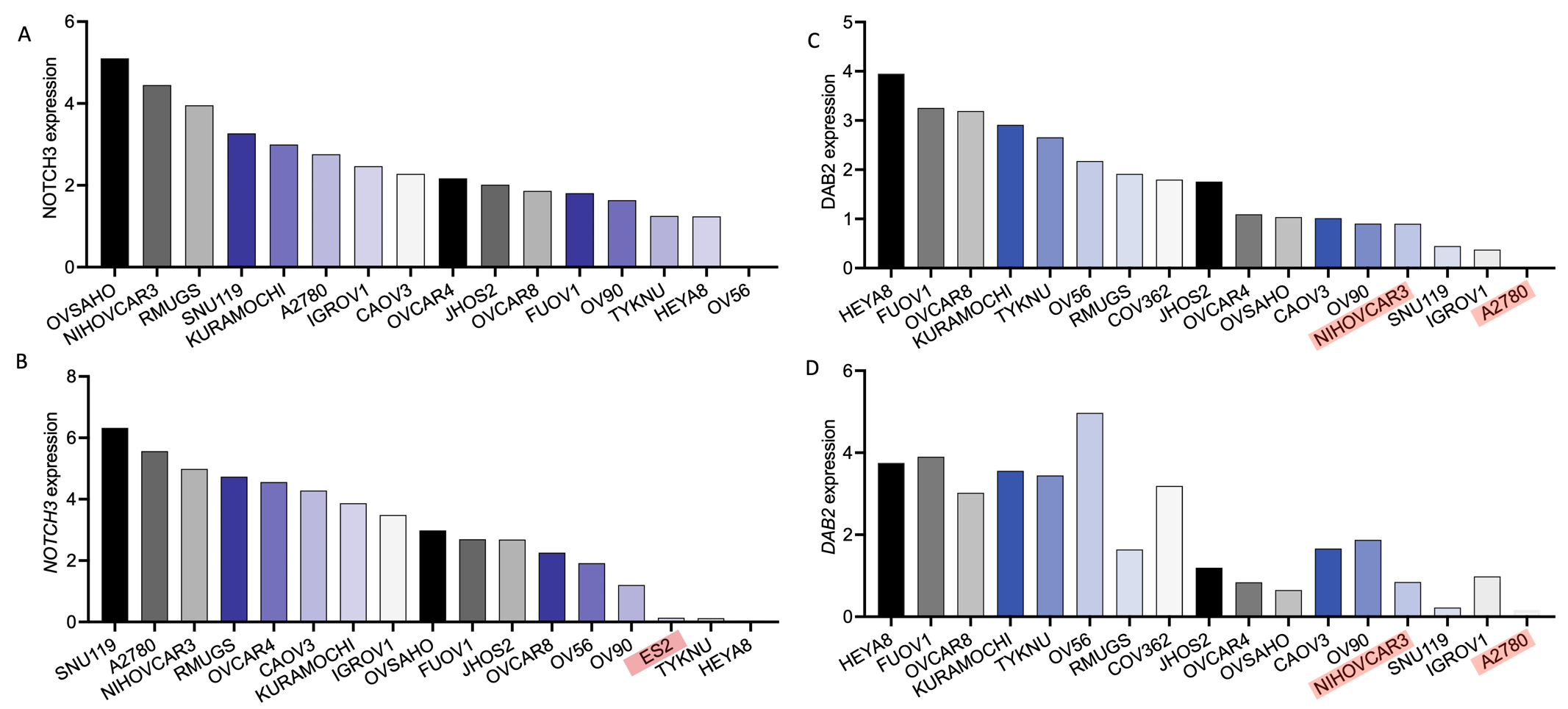
Supplementary Fig. 2: NOTCH3 and DAB2 protein and mRNA expression in ovarian cancer cell lines. NOTCH3 (A,B) and DAB2 (C,D) expression data from the Broad institute cancer cell line encyclopedia in ovarian cancer cell lines including (A,C) Proteomics (P98082) and (B,D) gene expression (expression public 22Q4).


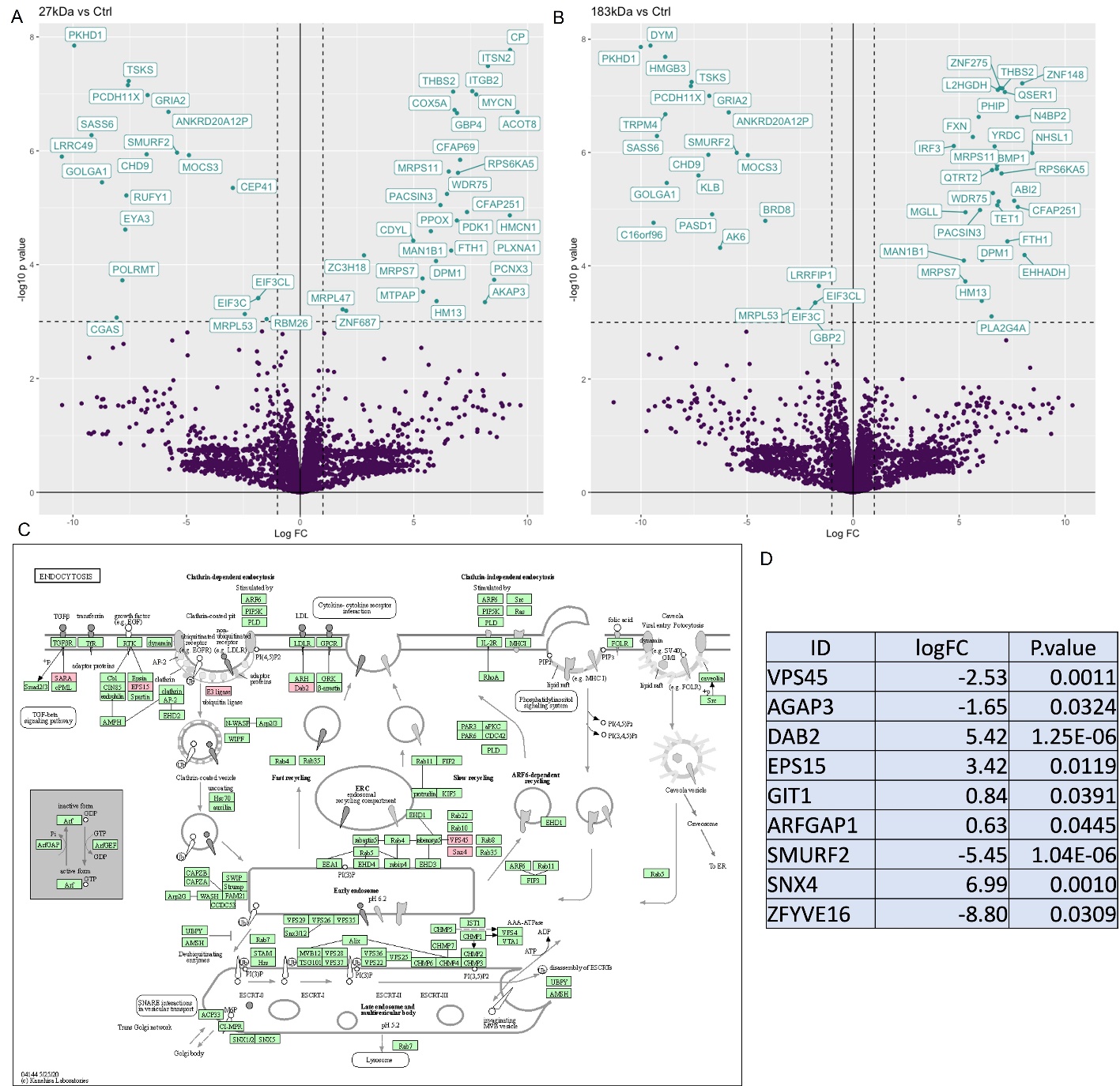


Supplementary Fig. 3. LC-MS:MS analysis of ES-2:ES-2-Rv-NICD3 (1:3) spheroids treated with Control (Ctrl), 27kDa, 183kDa and 1000kDa HA. Volcano plot of differentially expressed proteins in (A) 27kDa HA and (B) 183kDa HA vs Control (Ctrl) treated combination ES2:ES2-Rv-NICD3 (1:3) spheroids. (C) Kegg pathway analysis of endocytosis pathway proteins affected by 1000kDa HA treatment in ES2:ES2-Rv-NICD3 (1:3) spheroids highlighted in red. (D) log_2_ fold change (log_2_ FC) and p-values for proteins altered in the endocytosis pathway by 1000kDa HA.


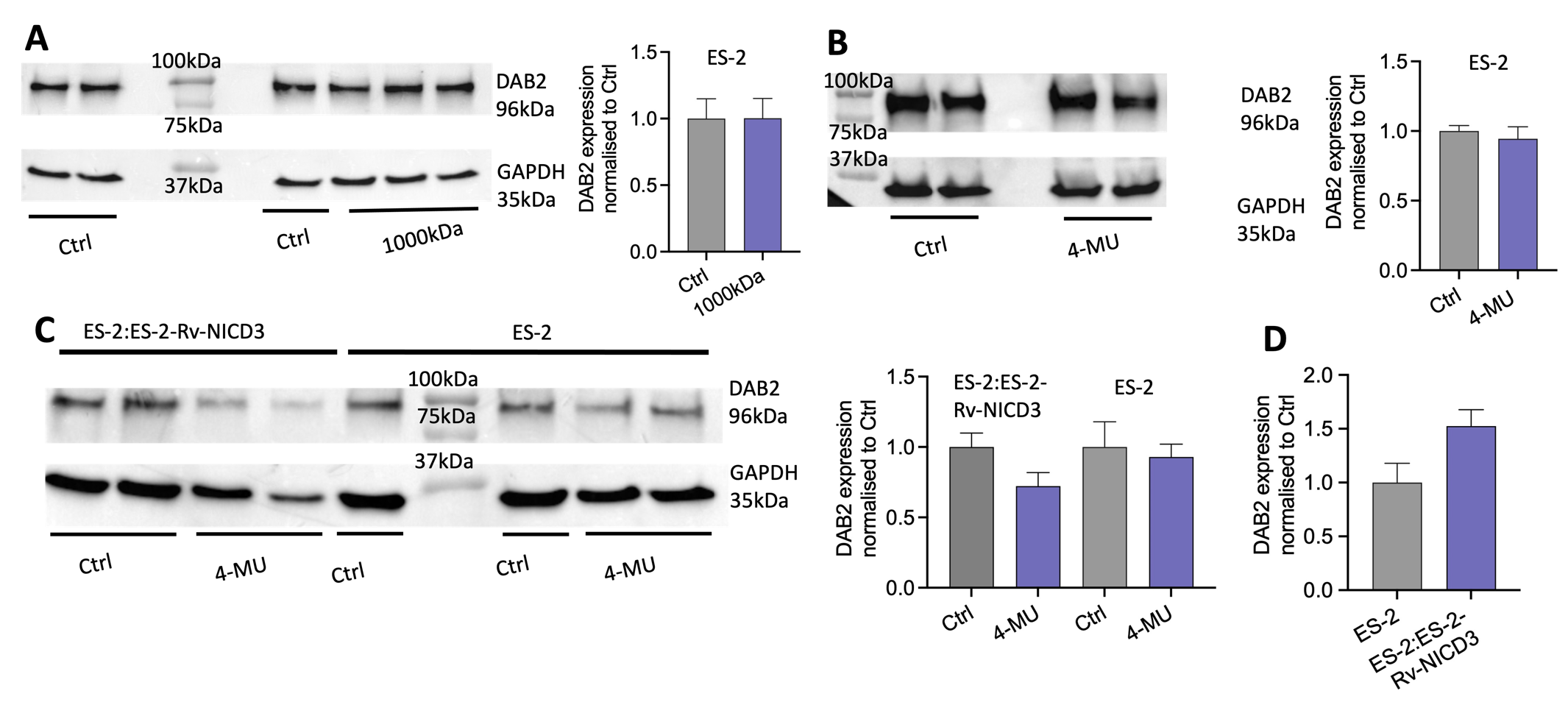


Supplementary Fig. 4. Effect of 1000kDa HA and HA synthesis inhibitor (4-MU) on DAB2 expression in wild type ES-2 and ES-2:ES-2-Rv-NICD3 ovarian cancer cells. Western blot analysis of DAB2 expression in (A) ES-2 spheroids treated with control (Ctrl) or 1000kDa HA (50µg/mL) in 24 well polyHEMA plates after 72hr (10,000 cells/well; n=3), (B) ES-2 monolayer cells treated with Ctrl or 4-MU (1mM) for 24hr and (C) ES-2 and ES-2:ES-2-Rv-NICD3 spheroids treated with Ctrl or 4-MU (1mM) for 24hr. (D) DAB2 expression in ES-2 and ES-2:ES-2-Rv-NICD3 ctrl treated spheroids (normalised to ES-2 WT). DAB2 expression (fold change) presented as mean ± SD.


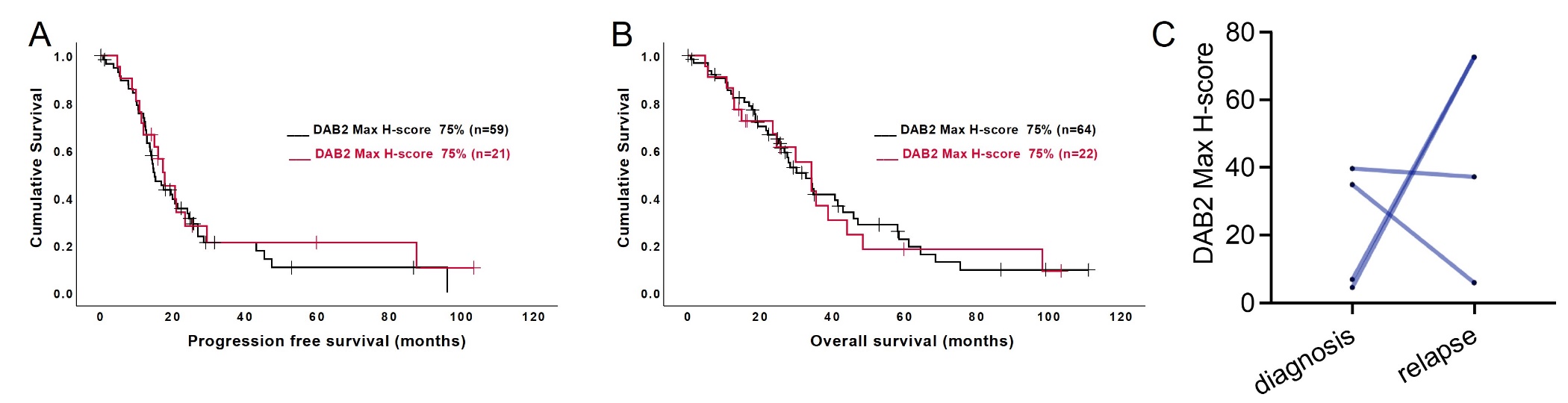


Supplementary Fig. 5. Epithelial *DAB2* expression is not associated with prognosis in HGSOC patients. TMA cohort was immunostained for DAB2 (Abcam, ab256524, 1/800). DAB2 immunostaining staining was quantitated using QuPath software and presented as H score. Kaplan-Meier survival plots for epithelial DAB2 H-score max in HGSOC tissues for (A) PFS (n=80, p=0.563) and (B) OS (n=86, p=0. 864). (C) Max H-score DAB2 epithelial staining in the matched HGSOC patient tissues at diagnosis and relapse (n=4)
